# Antidepressant Switching as a Proxy Phenotype for Drug Nonresponse: Investigating Clinical, Demographic, and Genetic Characteristics

**DOI:** 10.1016/j.bpsgos.2025.100502

**Published:** 2025-04-10

**Authors:** Chris Wai Hang Lo, Alexandra C. Gillett, Matthew H. Iveson, Michelle Kamp, Chiara Fabbri, Win Lee Edwin Wong, Dale Handley, Oliver Pain, Evangelos Vassos, Naomi R. Wray, Heather C. Whalley, Danyang Li, Allan H. Young, Andrew M. McIntosh, Cathryn M. Lewis

**Affiliations:** a Social, Genetic & Developmental Psychiatry Centre, Institute of Psychiatry, Psychology and Neuroscience, King’s College London, London, United Kingdom; b National Institute for Health Research Maudsley BRC at South London and Maudsley NHS Foundation Trust and King’s College London, London, United Kingdom; c Division of Psychiatry, Centre for Clinical Brain Sciences, University of Edinburgh, Edinburgh, United Kingdom; d Department of Biomedical and Neuromotor Sciences, University of Bologna, Bologna, Italy; e Department of Pharmacology, Yong Loo Lin School of Medicine, National University of Singapore, Singapore, Singapore; f Department of Basic and Clinical Neuroscience, Institute of Psychiatry, Psychology and Neuroscience, King’s College London, London, United Kingdom; g Institute for Molecular Bioscience, University of Queensland, Brisbane, Queensland, Australia; h Queensland Brain Institute, University of Queensland, Brisbane, Queensland, Australia; i Department of Psychiatry, University of Oxford, Oxford, United Kingdom; j Generation Scotland, Centre for Genomic and Experimental Medicine, Institute of Genetics and Cancer, University of Edinburgh, Edinburgh, United Kingdom; k Department of Psychological Medicine, Institute of Psychiatry, Psychology and Neuroscience, King’s College London, London, United Kingdom; l South London and Maudsley NHS Foundation Trust, London, United Kingdom; m Division of Psychiatry, Department of Brain Sciences, Imperial College London, London, United Kingdom; n Institute for Genomics and Cancer, University of Edinburgh, Edinburgh, United Kingdom; o Department of Medical & Molecular Genetics, King’s College London, London, United Kingdom

**Keywords:** Antidepressant response, Electronic health records, Generation Scotland, GWAS, Selective serotonin reuptake inhibitors (SSRIs), UK Biobank

## Abstract

**Background:**

Selective serotonin reuptake inhibitors (SSRIs) are a first-line pharmacological therapy in major depressive disorder (MDD), but treatment response rates are low. Clinical trials lack the power to study the genetic contribution to SSRI response. Real-world evidence from electronic health records provides larger sample sizes, but novel response definitions are needed to accurately define SSRI nonresponders.

**Methods:**

In the UK Biobank (UKB) (*N* = 38,813) and Generation Scotland (*N* = 1777) datasets, SSRI switching was defined using ≤90-day gap between prescriptions for an SSRI and another antidepressant in primary care. Nonswitchers were participants with ≥3 consecutive prescriptions for an SSRI. In the UKB, clinical, demographic, and polygenic score (PGS) associations with switching were determined, and the common-variant heritability was estimated.

**Results:**

In the UKB, 5133 (13.2%) SSRI switchers and 33,680 nonswitchers were defined. The mean time to switch was 28 days (interquartile range, 17–49). Switching patterns were consistent across the UKB and Generation Scotland (*n* = 498 switchers). Higher annual income and educational levels (odds ratio [OR] [95% CI] for a university degree, 0.73 [0.67–0.79] compared with no qualifications) were associated with lower levels of switching. PGSs for nonremission, based on clinical studies, were associated with increased risk of switching (OR, 1.07 [1.02–1.12], *p* = .007). MDD PGSs and family history of depression were not significantly associated with switching. Using genome-wide complex trait Bayesian, the single nucleotide polymorphism–based heritability was approximately 4% (SE 0.016) on the observed scale.

**Conclusions:**

This study identified SSRI switching as a proxy for nonresponse, scalable across biobanks with electronic health records, capturing demographics and genetics of treatment nonresponse, and independent of MDD genetics.

## Plain Language Summary

Genetics is a significant contributor to individual differences in antidepressant response. However, previous genetic studies using clinical trials have been severely underpowered to identify specific variants associated with treatment response. Leveraging electronic health records from UK Biobank and Generation Scotland, we identified switching from selective serotonin reuptake inhibitors (SSRIs), the first-line antidepressant, as a scalable proxy phenotype for nonresponse. Here, we described the switching patterns and explored demographic variables associated with switching, including socioeconomic status. We also demonstrated that SSRI switching was heritable, with a genetic component that correlated with nonresponse in clinical trials, but not with the genetics of depression susceptibility.

Worldwide, approximately 300 million people experience an episode of major depressive disorder (MDD) during their lifetime (1). Since the 1990s, selective serotonin reuptake inhibitors (SSRIs) have shown comparable efficacy for MDD treatment in primary care settings and have become one of the pharmacological treatment options together with tricyclic antidepressants (TCAs) (2). SSRIs have gained in popularity and become the first-line pharmacological treatment in MDD based on safety profiles (3,4). However, considerable variability exists in antidepressant response (5), with only about one-third of antidepressant users achieving clinical remission with their first prescribed antidepressant (6). Another one-third of patients go on to develop treatment-resistant depression (TRD), defined as the lack of response to 2 antidepressants with adequate duration and dosage (7). Identification of factors that predict response and nonresponse to antidepressants would enable personalized prescribing and improve treatment for MDD.

Multiple factors have been associated with response and nonresponse to antidepressant treatment. For example, childhood trauma is associated with poorer response to antidepressants (8), and in clinical trials, higher body mass index (BMI) and neuroticism scores have been significantly associated with antidepressant response (9,10). Patients with TRD often have higher depression symptom severity (11,12), and observational studies suggest that TRD is correlated with sociodemographic characteristics, such as unemployment (12). However, baseline MDD severity was not associated with symptom-level response in a meta-analysis of 91 clinical trials (13). Biomarkers for antidepressant response, including BDNF (brain-derived neurotrophic factor) (14), cortisol (8), and inflammatory markers (15), have shown inconsistent results.

Genetic factors have been associated with antidepressant response. Cytochrome P450 variants play a minor role in adverse events and response by affecting metabolism of antidepressants (16, 17, 18, 19). The largest genome-wide association study (GWAS) of clinical studies reported to date showed that antidepressant remission was heritable, with a single nucleotide polymorphism (SNP)–based heritability of up to 40% (20). However, genetic studies performed with clinical trials are underpowered to discover SNPs at genome-wide significance. Stringent inclusion criteria may also limit the generalizability of genetic findings to a population-wide level. Therefore, other study designs are required to increase the power to identify the genetic component of antidepressant response. Real-world data from electronic health records (EHRs) could fill this gap, because large sample sizes are available in biobanks with genetic data.

In EHRs, defining treatment response phenotypes is challenging, because response (or resistance) to antidepressant treatment is not directly coded in most records. Using clinical records, proxy phenotypes can be captured from unstructured text using natural language processing algorithms (21). Alternatively, phenotypes can be defined from structured prescription records, which are more readily available and scalable in population-wide biobanks. One feasible strategy is to capture switching events between antidepressants as an indication of nonresponse (22). This approach reflects clinical guidelines, according to which it is recommended that patients who fail to respond to an antidepressant switch to a different drug. Antidepressant switching in EHRs has been used to define TRD, where 2 switches occur within a single episode of depression (23). Antidepressant switching is also used in clinical trials as an alternative therapeutic strategy following inadequate response to the first antidepressant (often an SSRI) (24,25).

In this study, we used primary care prescribing records in the UK Biobank and dispensing records in Generation Scotland (26) to define a phenotype of switching from an SSRI to another antidepressant (of any class) during a single episode of depression. We characterized the prescription patterns and investigated the clinical, demographic, and polygenic predictors of SSRI switching. We also performed a GWAS of SSRI switching, showing that switching is heritable, and we propose switching as a proxy measure for nonresponse to SSRIs.

## Methods and Materials

### Primary Sample—the UKB

**Figure 1:**
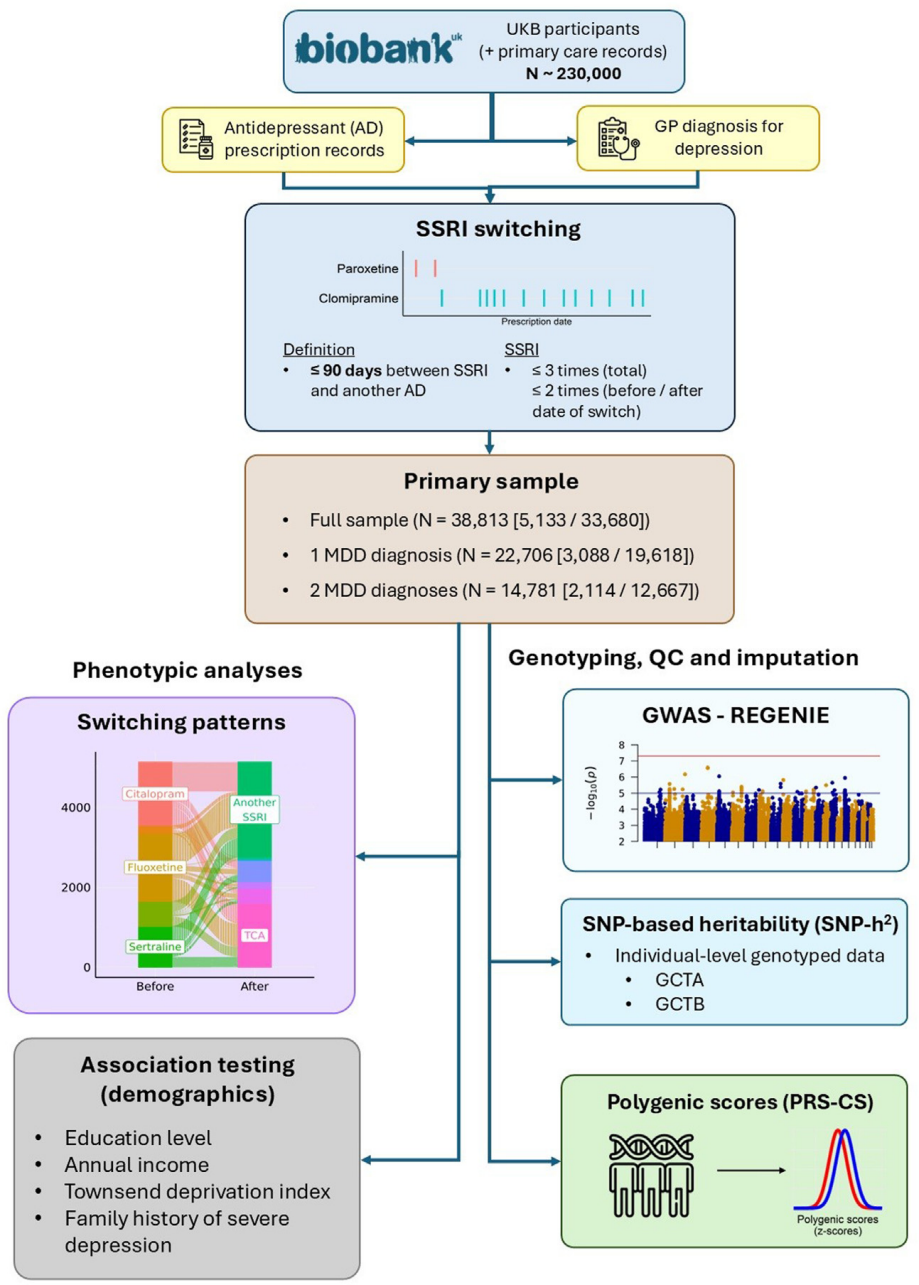
Study design overview for the primary sample (UKB). GCTA, genome-wide complex trait analysis; GCTB, genome-wide complex trait Bayesian; GP, general practitioner; GWAS, genome-wide association study; MDD, major depressive disorder; QC, quality control; SNP, single nucleotide polymorphism; SSRI, selective serotonin reuptake inhibitor; UKB, UK Biobank.

The UKB is a prospective health study that recruited over 500,000 volunteers aged 40 to 69 years in the United Kingdom from 2007 to 2010 (27,28). Genome-wide genotyping, available for all UKB participants, underwent standard quality control (QC) and imputation. Further description of UKB samples, as well as details on genotyping QC and imputation, is available in Supplemental Methods, and an analysis flowchart is shown in Figure 1. Linkage with primary care data is available for ∼230,000 UKB participants, containing clinical events (coded by READ v2 or CTV-3) and prescription records (coded by READ v2, READ v3, BNF, or dm+d) (29). READ v3 diagnosis codes used are listed in Table S1, with antidepressants and mapped drug classes listed in Table S2.

Patients with depression were identified using primary care diagnosis records for depressive disorders, using a previously validated algorithm (23). Participants with any primary care diagnosis for bipolar disorder, psychosis, or substance abuse were excluded. Not all patients prescribed SSRIs had a depression diagnostic code assigned. To examine the impact of diagnosis, we analyzed 3 datasets of participants prescribed an SSRI: 1) all patients satisfying the switcher/nonswitcher criteria regardless of depression diagnoses; 2) patients with ≥1 depression diagnostic record; and 3) patients with ≥2 depression diagnostic records.

Sociodemographic and clinical variables extracted from the UKB were self-reported sex (field ID 31-0.0), educational qualifications (field ID 6138-0.0, with “none of the above” as reference level), annual income (as average total household income before tax, field ID 738-0.0, with “less than £18,000” as reference level), Townsend Deprivation Index (field ID 189-0.0), BMI (field 21001-0.0), and family history of depression (with “no” as reference level). A positive family history of depression was defined when at least 1 parent (field ID 20107-0.0 and 20110-0.0) or sibling (field ID 20111-0.0) reported severe depression. Polygenic scores (PGSs) for MDD (UKB participants removed) (30), schizophrenia (31), and antidepressant nonremission (20) were computed using PRS-CS (32) with the GenoPred pipeline (version 1) (33,34). Details of polygenic scoring methods and GWAS summary statistics are summarized in Supplemental Methods and Table S3.

### Replication Sample—Generation Scotland

Replication analyses were performed in Generation Scotland to compare switching patterns across health care practices. Generation Scotland is a family-based longitudinal study which recruited over 24,000 volunteers from 2006 to 2011, with information on demographic variables and physical and mental health measurements (26). Linkage to dispensed prescriptions in primary care was available for over 90% of participants (26). Details on the Generation Scotland replication sample are summarized in Supplemental Methods.

### Phenotype Definition: SSRI Switching

SSRI switchers were defined as participants who were prescribed an SSRI and who then received a prescription for another antidepressant within a 90-day window, from 5 to 95 days after the initial prescription (a switching event). The following additional criteria were applied:

1. A minimum 5-day window between prescriptions to avoid capturing overlapping prescriptions of 2 antidepressants (augmentation) as switches;
2. The preswitch SSRI was prescribed ≤3 times during the entire course of the prescribing history to ensure that it was not prescribed in future treatment episodes;
3. The preswitch SSRI was prescribed ≤2 times before the switch date to capture early switchers specifically; and
4. The preswitch SSRI was prescribed ≤2 times after the switch date, to ensure that augmentation was not captured, while giving a brief allowance period for cross-tapering.

SSRI nonswitchers were defined as patients who did not switch from any SSRIs and who received ≥3 prescriptions for an SSRI.

The SSRI index date was defined as the first prescription date for the SSRI in switchers and nonswitchers. A schematic figure of definitions of switchers and nonswitchers is shown in Figure S1.

### Analysis of Switching Patterns, Clinical and Demographic Variables, and PGSs

Descriptive analyses were performed on SSRI switchers and nonswitchers by index SSRI drug, drug class postswitch, time to switch, and age at index date. Differences in the distributions of the variables between SSRI switchers and nonswitchers were assessed by nonparametric statistical tests, including Pearson’s χ^2^ test (for binary variables), Kruskal-Wallis rank-sum test (for categorical variables of more than 2 levels), and Wilcoxon rank-sum test (for continuous variables).

Associations between switching and sociodemographic variables at baseline assessment and PGSs were tested by logistic regression, with models adjusted for self-reported sex (with female as the reference level), index date of SSRI, and assessment center (with Centre_10003 as reference level). Associations with PGSs were further adjusted for 10 principal components for population stratification. For related individuals, 1 participant was removed based on third-degree relatedness (kinship coefficient < 0.044) by greedy matching, with switchers being preferentially retained. Statistical significance was assessed by likelihood ratio test and corrected for multiple testing by Bonferroni correction within each sample (*p* ≤ .0071; correcting for 4 sociodemographic variables [educational qualifications, annual income, family history of severe depression, and Townsend Deprivation Index] and 3 PGSs [nonremission, MDD, SCZ]).

### Genome-Wide Association Study

Genome-wide association analysis was performed on SSRI switching using REGENIE (35), a 2-step software program for genome-wide analysis. Given the low ratio of switchers to nonswitchers, SNPs with low allele frequencies may go into quasi-complete separation when standard logistic regression models are applied in GWASs (35). Therefore, SNP effect sizes underwent Firth correction to control for false positives as recommended in REGENIE documentation (35).

For genetic analyses, we tested for differences in the distributions of assessment center and genotyping batch between switchers and nonswitchers using Kruskal-Wallis rank-sum tests to assess which covariates should be included (see Table S9). Covariates included in the GWASs were SSRI index date, genetic sex, assessment center, and 10 principal components for population stratification.

### SNP-Based Heritability Estimation

SNP-based heritability (*h*^2^_SNP_) was estimated using 2 genomic relatedness–based restricted maximum likelihood (GREML)–based methods, genome-wide complex trait analysis (GCTA) (36) and genome-wide complex trait Bayesian (GCTB) (37). *h*^2^_SNP_ was reported on the observed scale because the sample was unselected for SSRI treatment. In GCTB, the *h*^2^_SNP_ estimates were constrained to be between 0 and 1 in each iteration, and the distributions of *h*^2^_SNP_ estimates can be skewed when the true *h*^2^_SNP_ is close to 0. To strengthen the robustness of our findings, the posterior mode and 95% highest posterior density (HPD) credible intervals were also reported for GCTB. The degree of polygenicity (*Pi*) and negative selection (*S*) were also reported. Full details of *h*^2^_SNP_ estimation are available in Supplemental Methods.

## Results

### Descriptive Analyses

**Figure 2:**
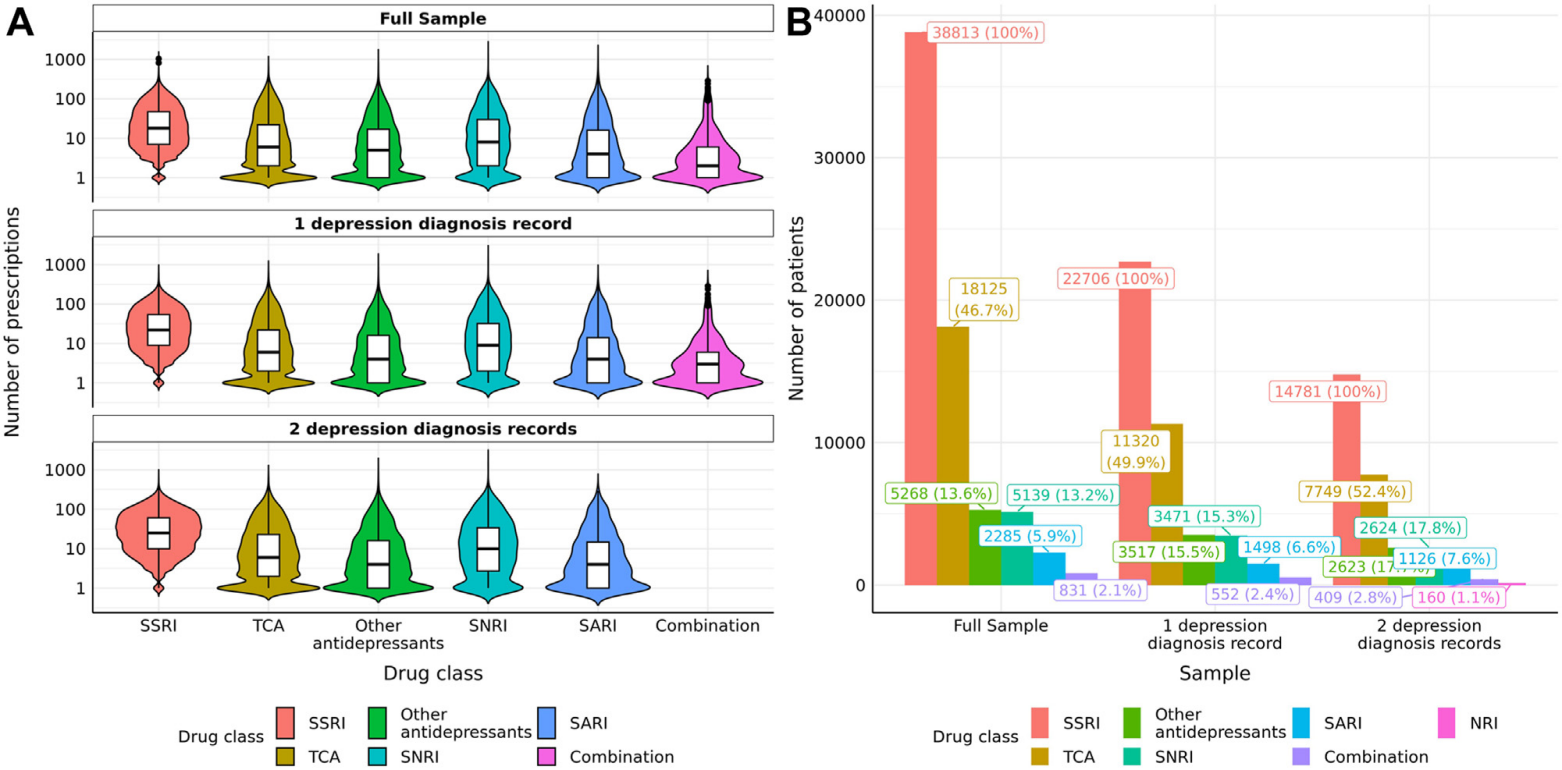
Number of antidepressant prescriptions in the primary sample (UK Biobank). **(A)** Number (median [interquartile range]) of prescriptions of primary sample (by drug classes with number of patients >500); **(B)** proportion of patients receiving at least 1 prescription for a particular drug class. Only drug classes consisting of >1% of sample sizes were labeled. Details on the statistics are available in the Supplement. MAOI, monoamine oxidase inhibitor; NRI, norepinephrine reuptake inhibitor; SARI, serotonin antagonist and reuptake inhibitor; SNRI, serotonin-norepinephrine reuptake inhibitor; SSRI, selective serotonin reuptake inhibitor; TCA, tricyclic antidepressant.

In the UKB, a total of 5133 SSRI switchers and 33,680 nonswitchers were identified from prescription records (full sample). Baseline characteristics of SSRI switchers and nonswitchers are given in Table 1 and Figure S2. Patients in the total sample had a median of 18 SSRI prescriptions (interquartile range [IQR], 7–47) (Figure 2 and Table S4) across all prescribing history, primarily spanning from the 1990s to 2018 (Figure S3). Of these participants, 3088 (60%) switchers and 19,618 (58%) nonswitchers had at least 1 diagnostic record for MDD, with 2114 (41%) switchers and 12,667 (38%) nonswitchers having at least 2 MDD diagnostic records (Figure S2). In the total sample, 67% were female, and 96% were of White ethnicity (Table 1). Approximately one half of participants had at least 1 prescription for a TCA (total sample: *n* = 18,125 [46.7%]; 1 depression record: *n* = 11,320 [49.9%]; and 2 depression records: *n* = 7749 [52.4%]) (Table S5); and 13% to 18% received ≥1 prescription for a serotonin-norepinephrine reuptake inhibitor (Table S5). In Generation Scotland, a total of 498 SSRI switchers and 1279 nonswitchers with at least 1 diagnostic record for depression were identified (Table S7).

**Table 1:** Primary Sample (UK Biobank) Summary

Characteristic	Nonswitchers, *n* = 33,680	Switchers, *n* = 5133	*p* Value^a^
Index SSRI			<.001
Fluoxetine	11,278 (33%)	1687 (33%)	
Paroxetine	4125 (12%)	621 (12%)	
Citalopram	12,448 (37%)	1586 (31%)	
Sertraline	4664 (14%)	1024 (20%)	
Escitalopram	1105 (3.3%)	201 (3.9%)	
Fluvoxamine	60 (0.2%)	14 (0.3%)	
Age on Index Date, Years	53 (46–59)	54 (47–61)	<.001
Time to First Switch, Days	–	28 (17–49)	
Sex			.7
Female	22,598 (67%)	3428 (67%)	
Male	11,082 (33%)	1705 (33%)	
Ethnic Background			.016
Asian	422 (1.3%)	89 (1.7%)	
Black	237 (0.7%)	38 (0.7%)	
Mixed	198 (0.6%)	20 (0.4%)	
White	32,425 (96%)	4906 (96%)	
Not available	398 (1.2%)	80 (1.6%)	
Body Mass Index	27.4 (24.6–31.1)	27.1 (24.2–30.7)	<.001
Unknown	249	47	
Neuroticism Score	6.0 (4.0–9.0)	7.0 (4.0–10.0)	<.001
Unknown	7479	1184	
Family History for Severe Depression	6977 (21%)	1108 (22%)	.2
Qualifications			<.001
None of the above	6722 (20%)	1223 (24%)	
Secondary	9689 (29%)	1475 (29%)	
Vocational	3912 (12%)	628 (12%)	
Further	3734 (11%)	523 (10%)	
University degree	9183 (27%)	1214 (24%)	
Not available	440 (1.3%)	70 (1.4%)	
Annual Income, £			<.001
<18,000	8813 (26%)	1536 (30%)	
18,000–30,999	7519 (22%)	1141 (22%)	
31,000–51,999	7088 (21%)	964 (19%)	
52,000–100,000	4445 (13%)	550 (11%)	
>100,000	816 (2.4%)	95 (1.9%)	
Not available	4999 (15%)	847 (17%)	
TDI	−1.8 (−3.4 to 1.1)	−1.8 (−3.4 to 1.1)	.6
Unknown	50	6	

Values are presented as *n*, *n* (%), or median (interquartile range).

SSRI, selective serotonin reuptake inhibitor; TDI, Townsend Deprivation Index.

a Kruskal-Wallis rank-sum test, Wilcoxon rank-sum test, or Pearson’s χ^2^ test.

### Patterns of SSRI Switching

In both UKB and Generation Scotland, the most prescribed/dispensed SSRI antidepressants were fluoxetine and citalopram. Around one-third received fluoxetine (UKB: *n* = 1687 [33%] switchers and 11,278 [33%] nonswitchers) (Table 1) (Generation Scotland: *n* = 165 [33%] switchers and 433 [34%] nonswitchers) (Table S7). Another one-third received citalopram (UKB: *n* = 1586 [31%] switchers and 12,448 [37%] nonswitchers; Generation Scotland: *n* = 190 [38%] switchers and 604 [47%] nonswitchers). Paroxetine and sertraline were also commonly prescribed/dispensed, each to over 10% of participants.

**Figure 3:**
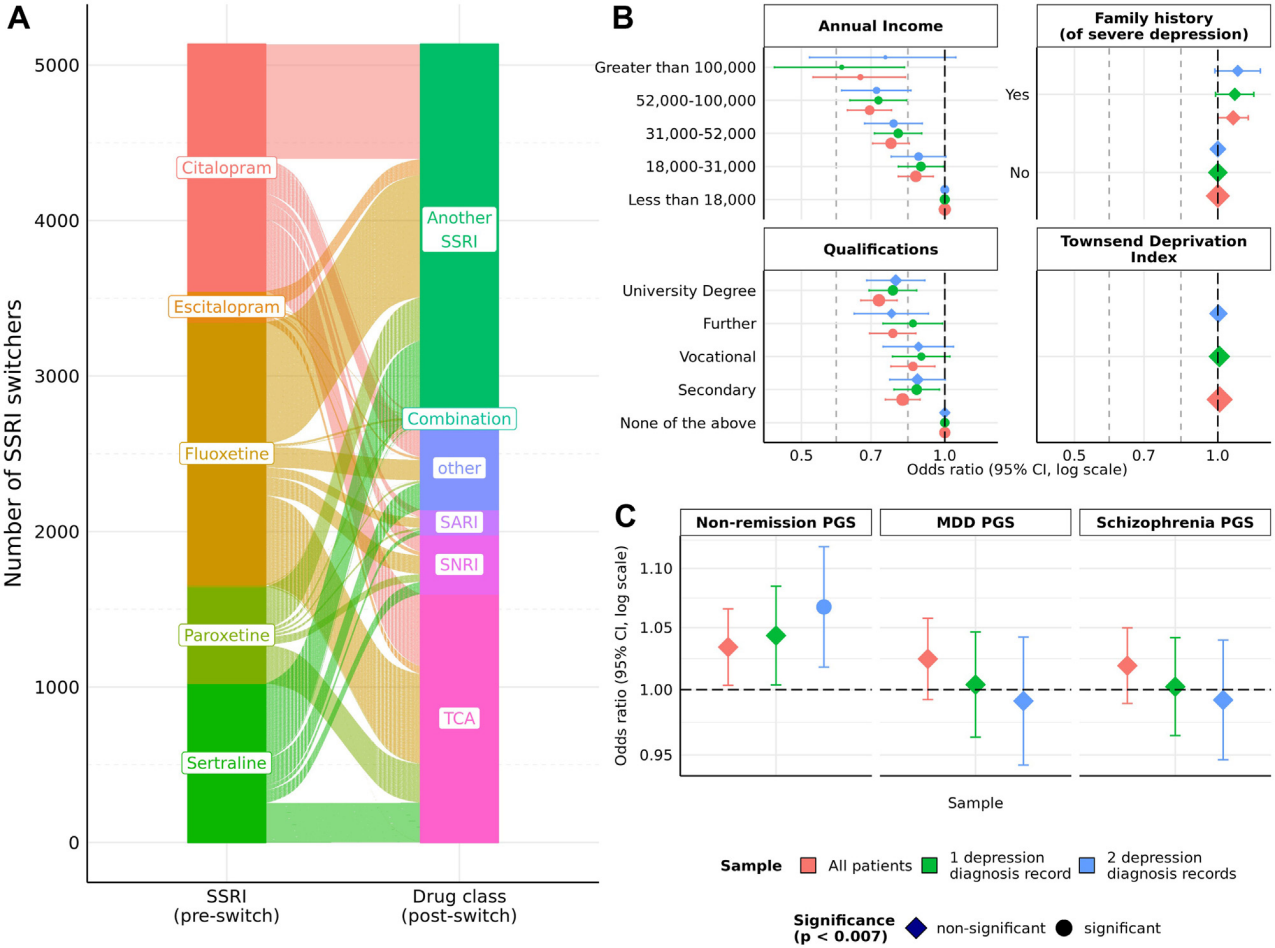
Switching patterns and association testing with clinical and sociodemographic variables and polygenic scores in the primary sample (UK Biobank). **(A)** SSRI switching patterns in the UK Biobank, stratified by index SSRI and drug class after switch; **(B)** association testing between demographic variables and SSRI switcher statuses; **(C)** association testing between polygenic scores and SSRI switcher status. Only SSRIs (preswitch) and drug classes (postswitch) with at least a sample size of *n* = 70 are labeled. MDD, major depressive disorder; PGS, polygenic score; SARI, serotonin antagonist and reuptake inhibitor; SNRI, serotonin-norepinephrine reuptake inhibitor; SSRI, selective serotonin reuptake inhibitor; TCA, tricyclic antidepressant.

Most SSRI switches occurred within 6 weeks of the index prescription in both the UKB (median time to switch in days [IQR], 28 [17–49]) (Table 1) and Generation Scotland (31 [31–61]) (Table S7). Distributions of time to switch were similar across index SSRIs in the UKB (Figure S4). The proportion of switchers and time to switch were generally comparable across males and females (Figure S6). In the UKB, approximately one half of switching events were to another SSRI (*n* = 2380; 46%) or a TCA (*n* = 1597; 31%) (Figure 3 and Table S8). Similar patterns were observed in Generation Scotland, with one half switching to another SSRI (*n* = 237; 47.6%) and fewer patients changing to a TCA after switching (*n* = 73; 14.7%) (Figures S7 and S9).

Across the 22 assessment centers in the UKB, the proportions of switchers differed significantly, varying between 9% and 19% (*p* = 2.5 × 10^−8^) (Figure S8 and Table S10). The rate of switching increased with more recent index dates (Pearson correlation coefficient between switching rate and time [*r*] = 0.43, *p* = 1.0 × 10^−5^), and the period between the index date and switching was shorter (*r* = −0.42, *p* = 1.5 × 10^−5^) (Figure S9).

### Associations With Sociodemographic Variables and PGSs

In UKB, higher educational levels were associated with lower odds of SSRI switching (odds ratio [OR] for university degree [95% CI], 0.73 [0.67–0.79]; *p* = 1.53 × 10^−10^ [compared with the reference group of no qualifications]) (Table S11). The results were consistent when samples were limited to at least 2 MDD diagnoses in primary care (0.79 [0.69–0.91], *p* = .013) and at least 1 MDD diagnosis (0.78 [0.69–0.87], *p* = .001). Similar findings were observed for annual income, with higher income associated with lower risks of SSRI switching for annual income >£100,000 compared with the reference group of <£18,000 in the total sample (0.66 [0.53–0.83], *p* = 6.79 × 10^−15^). Effect sizes were similar when constrained to at least 2 MDD diagnosis records (0.75 [0.52–1.06], *p* = 1.43 × 10^−4^) and 1 MDD diagnosis record (0.61 [0.44–0.82], *p* = 4.92 × 10^−7^). SSRI switching was not associated with the Townsend Deprivation Index, a measurement of material deprivation.

Family history of severe depression was only nominally associated with SSRI switching status in the total sample (1.08 [1.00–1.16], *p* = .048) and was not associated with switching in patients with 1 (1.09 [0.99–1.19], *p* = .084) or 2 (1.10 [0.99–1.23], *p* = .088) MDD diagnosis records. The PGS for MDD was not associated with SSRI switching (1.02 [0.99–1.06], *p* = .138 [for the total sample]). Similar results were found when samples were restricted to participants with at least 1 (1.00 [0.96–1.05], *p* = .848) and 2 (0.99 [0.94–1.04], *p* = .73) MDD diagnostic records.

Higher PGS for antidepressant nonremission was associated with an increased risk of SSRI switching, with modest effect sizes. Only the associations in patients with 2 MDD diagnostic records survived multiple testing correction (1.07 [1.02–1.12], *p* = .007). The associations were nominally significant in the total sample (1.03 [1.00–1.07], *p* = .029) and in participants with a single MDD diagnostic record (1.04 [1.00–1.08], *p* = .031), with similar directions of association (Figure 3 and Table S11).

### Genetic Analyses

A GWAS was performed using REGENIE on 4773 SSRI switchers and 31,561 nonswitchers in the total sample. The sample sizes were reduced to 2868 SSRI switchers and 18,360 nonswitchers for 1 MDD diagnosis record and to 1967 switchers and 11,853 nonswitchers for 2 MDD diagnosis records. No variants were identified in either analysis at genome-wide significance (*p* < 5 × 10^−8^), with Manhattan plots shown in Figures S15 to S17. At a suggestive significance threshold (*p* < 1 × 10^−5^), 21, 25, and 30 independent SNPs were identified with the total sample, as well as samples with 1 or 2 MDD diagnoses, respectively.

**Figure 4:**
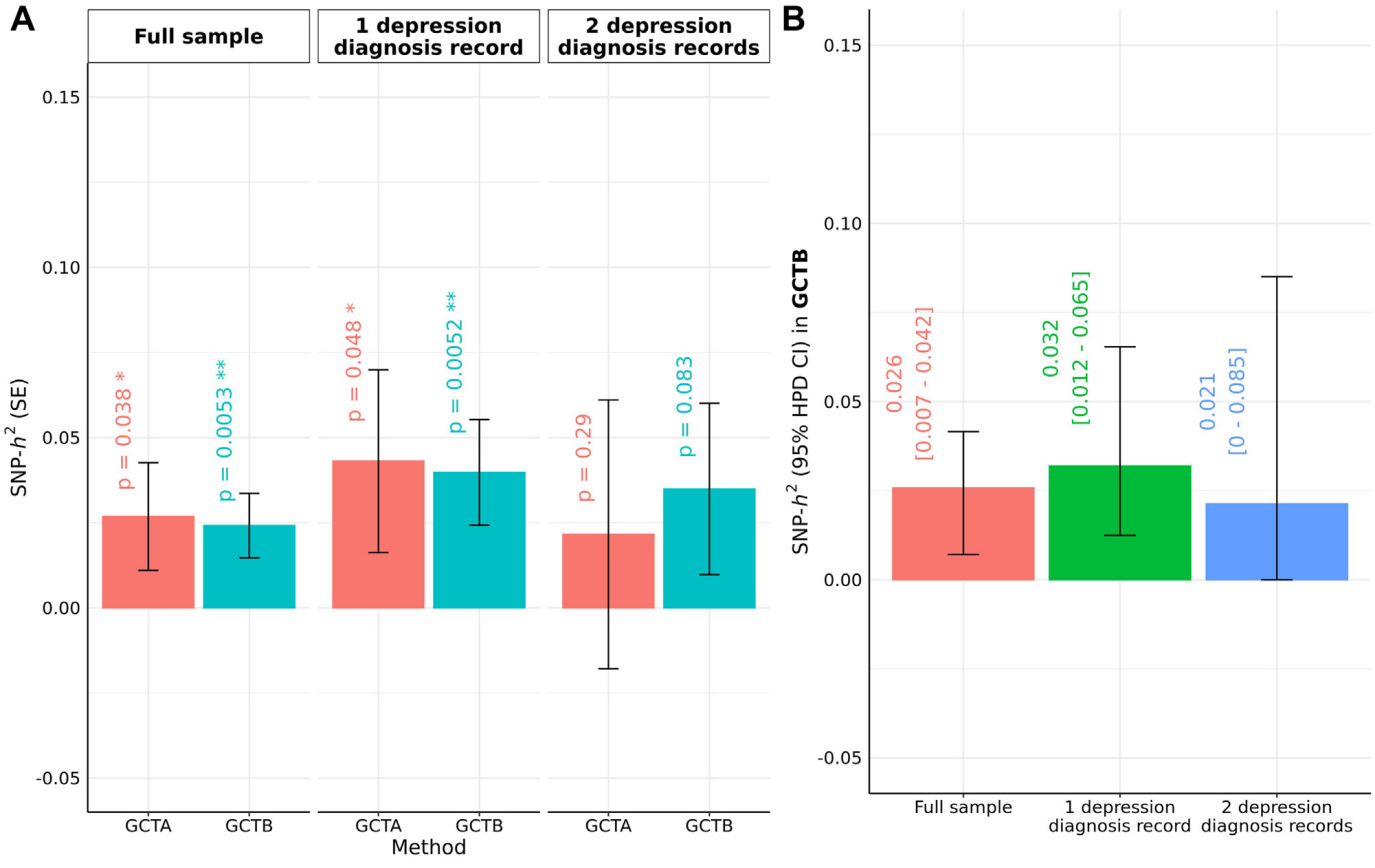
*h*^2^_SNP_ estimates for SSRI switching by GCTA and GCTB. *h*^2^_SNP_ on observed scale stratified by number of depression diagnoses in primary sample, expressed in **(A)** posterior mean (SE) and **(B)** posterior mode (95% HPD credible intervals) for GCTB. ∗*p* < .05, ∗∗*p* < .01. GCTA, genome-wide complex trait analysis; GCTB, genome-wide complex trait Bayesian; *h*^2^_SNP_, SNP-based heritability; HPD, highest posterior probability; SNP, single nucleotide polymorphism; SSRI, selective serotonin reuptake inhibitor.

SSRI switching had an *h*^2^_SNP_ significantly different from 0 on the observed scale in the total sample (GCTA *h*^2^_SNP_ [SE]: 0.0268 [0.016], *p* = .038; GCTB 0.0242 [0.01], *p* = .005), as well as participants with 1 MDD diagnosis record (GCTA 0.0431 [0.0268], *p* = .048; GCTB 0.0398 [0.016], *p* = .005) (Figure 4 and Table S12). The posterior mode for *h*^2^_SNP_ in GCTB was also different from 0 (0.032; 95% HPD credible intervals, 0.012–0.065). *h*^2^_SNP_ estimates were nonsignificant for patients with at least 2 MDD diagnoses records in GCTB (0.035 [0.025], *p* = .08) and GCTA (0.0216 [0.0394], *p* = .293).

## Discussion

EHRs offer promising opportunities to define antidepressant response outcomes from prescribing patterns, but these outcomes have not been well characterized to date. In this study, we used SSRI switching as a proxy phenotype for nonresponse to antidepressants. The measure reflects the current clinical practice of moving patients from first-line SSRI treatment to a different antidepressant in cases of no or poor response (3,38), the signs of which could be evident from 2 to 4 weeks at the earliest (39). We identified associations of switching with demographic and genetic profiles and showed a modest heritability for the switching phenotype.

Our phenotypic definition of switching in the UKB aimed to capture antidepressant switchers, following previous work on more than 260,000 participants prescribed antidepressants in the UK Clinical Practice Research Data Link (CPRD) database of primary care records (40). In the CPRD study, most SSRI users switched to another SSRI as second-line therapy (54.1%) (40), which is consistent with our findings. In the CPRD, 9.3% of antidepressant users switched, with a median time to switch of 45 days (40). In comparison, SSRI users in the UKB had a higher proportion of switching (13.2%) and a shorter time to switch (median, 28 days). The study period for the CPRD study was from 2005 (40), compared with the 1990s in the UKB, with the latter being the period when SSRIs became the first-line therapy in clinical guidelines (2).

We used a 90-day window between prescription dates of 2 different antidepressants to capture switching events. This contrasts with the shorter window applied in the CPRD (40), where switching events were identified from a 30-day or less gap between the expected end dates of the first treatment and the start date of the second treatment. Using longer windows allowed us to capture more switchers for genetic analyses, but it is less specific to the exact cause of switching in the samples. Notably, using different window lengths did not substantially alter effect sizes for associations between switching and CYP2C19 metabolizer status in a previous UKB analysis (22). Our definition of switching events primarily relies on prescription dates of different antidepressants. This avoids making inferences of treatment duration where it cannot be accurately estimated from dosage instructions and quantity of prescriptions, as in UKB EHR data.

SSRI switching captures demographic and clinical variables associated with nonresponse to antidepressants. We showed that the proportions of participants who switched were lower among those with higher incomes and higher educational levels, which is consistent with evidence that higher PGSs for education attainment were associated with remission in clinical trials (20). Higher levels of antidepressant response have been associated with higher socioeconomic status (SES) in a systematic review of clinical trials (41) and in a Nordic registry study (42). It was suggested previously that SES may directly contribute to poor prognosis if it is causal to the development of depression itself (43). SES is also correlated with access to treatment and mental health services, which may in turn affect adherence to antidepressant and treatment outcomes (41). Our results confirm that SSRI switching in EHRs shows sociodemographic profiles similar to those seen in antidepressant nonresponse in trials and retrospective clinical studies.

Results from the genetic analyses also support SSRI switching as a proxy phenotype for antidepressant nonresponse. SSRI switching in the UKB was associated with a PGS for nonremission but not with an MDD PGS or with a family history of depression. These results indicate that the genetics of SSRI switching overlaps with the genetics of antidepressant response in clinical trials but is independent of the genetics of MDD (20). MDD PGSs capture the genetics of susceptibility and of symptom severity (44,45), a strong predictor of antidepressant nonresponse. However, these genetic factors were not correlated with response outcomes in our analysis. Mixed evidence has been found in previous studies, with positive correlations between MDD PGSs and poorer response in smaller clinical trials (16,46,47), but none was robust to multiple testing. Our genome-wide analysis of SSRI switching was underpowered to detect specific risk variants, but the identification of this EHR-based phenotype for antidepressant response/nonresponse opens opportunities for expanding the sample size in other real-world data sources. We sought replication of the UKB SSRI switching phenotype in Generation Scotland, which has 20,000 participants. However, with only 1777 study members classified as SSRI switchers or nonswitchers, only a limited analysis could be performed. We reported the results here for completeness.

Genetic analyses in the UKB revealed a modest heritable signal for SSRI switching (*h*^2^_SNP_ = ∼0.04). Antidepressant response was also found to be significantly heritable in a GWAS meta-analysis of clinical trials (20), with estimates of *h*^2^_SNP_ of 0.083 (SE 0.035) on the observed scale. The lower *h*^2^_SNP_ estimates for SSRI switching likely occur because switching is a proxy phenotype that captures nonresponse alongside adverse events and other features of SSRI treatment. Although genetic correlations cannot be calculated due to limited sample sizes, the positive association between the nonremission PGS and SSRI switching confirms a common genetic component for these phenotypes. Our genetic heritability estimates differed between the GREML-based methods of GCTA and GCTB and stratifying by 1 or 2 depression diagnosis records. The *h*^2^_SNP_ estimates with 2 MDD diagnosis records were not significantly different from 0, meaning that we were unable to consistently confirm nonzero heritability across samples, likely due to heterogeneity and insufficient statistical power. However, our results highlight the potential of using switching as a proxy phenotype to capture nonresponse to antidepressants, which is scalable across EHR data resources (26,48) and will allow future meta-analyses to obtain more robust estimates.

Although SSRI switching appears to capture dimensions of nonresponse phenotypically and genetically, it is important to be aware of the heterogeneity in the exact causes of switching in EHRs. Patients with MDD may switch antidepressants for a multitude of reasons, including lack of efficacy, noncompliance to treatment, and side effects (38). Using dosage information in prescription records would enable additional investigation of the causes of switching, but such data are not readily available in UKB prescription records. Stratifying switchers by switching windows may be another feasible strategy, because patients switching earlier are more likely to reflect tolerability issues, while later switchers may switch due to a lack of efficacy.

This study had several limitations in data availability and sample sizes in the UKB primary care records. Firstly, nonremission PGSs were calculated using genetic studies in antidepressant clinical trials with moderate sample sizes. Using the UKB sample alone, genetic analyses were also underpowered and were at the margins of requirements for robust *h*^2^_SNP_ estimates. Larger sample sizes are necessary to replicate the current findings. In GWASs of mental disorders, broadening phenotypic definition increases the power to detect associated loci but reduces specificity (49,50). We decided to maintain a balance between the 2 by stratifying SSRI switchers with at least 1 or 2 MDD diagnostic records in primary care to ensure that the antidepressant had been prescribed for depression instead of for conditions such as anxiety and insomnia (51). However, we did not limit the samples to requiring primary care diagnoses of depression on or before SSRI exposure. As a check, primary care diagnoses of depression preceded index SSRI exposure in over 70% of participants in our samples (Table S6), despite the possible missingness of diagnoses records in EHRs. While the UKB has rich EHR data on prescribing, it lacks the response measures available in clinical trials, including depression symptom scores at baseline and during treatment. Finally, the choice of treatment and choice to switch treatment have multiple contributing factors, which our analyses might not have fully accounted for, with the possibility of residual confounding.

### Conclusions

Using primary care records in the UKB dataset, we characterized SSRI switching as a data-driven proxy that captures clinical, demographic, and genetic dimensions of SSRI nonresponse. Switching patterns identified in our phenotyping algorithm were consistent with current clinical practice, with most patients switching to another SSRI. SSRI switching captured the genetics of antidepressant response and was distinct from the genetics of MDD susceptibility. We also identified modest but significant heritability for SSRI switching. In summary, SSRI switching defined from EHRs is a valuable phenotype to study the genetics of SSRI response. As a highly scalable phenotype, SSRI switching can contribute to pharmacogenetic research moving toward personalized prescribing.

## Supplementary Material

Supplemental Methods, Figures S1–S20, and Tables S1–S15

Key Resources Table
