# Supplementary material for: Antidepressant Switching as a Proxy Phenotype for Drug Nonresponse: Investigating Clinical, Demographic, and Genetic Characteristics
Source: Biol Psychiatry Glob Open Sci. 2025 Apr 10;5(4):100502. doi: 10.1016/j.bpsgos.2025.100502 (PMC12162021; doi:10.1016/j.bpsgos.2025.100502)
Supplement: Supplemental Methods, Figures S1–S20, and Tables S1–S15 [file mmc1.pdf]

SUPPLEMENTARY INFORMATION

Antidepressant Switching as a Proxy Phenotype for Drug Non-Response: Investigating Clinical, Demographic, and Genetic Characteristics

Lo *et al.*

Table of Contents

Supplementary Methods 4

Primary Sample – UK Biobank (UKB) 4

Sample description 4

Genotyping 4

Primary care records 5

Replication Sample – Generation Scotland 6

Sample description 6

Polygenic scoring 7

Genome-wide association studies (GWASs) 8

SNP-based heritability (h^2_{SNP}) estimation 8

Supplementary tables 10

Supplementary table 1. READ v3 diagnosis codes used in this study 10

Supplementary table 2. Antidepressant names and respective drug classes 12

Supplementary table 3. GWAS summary statistics used for polygenic scoring 14

Supplementary table 4. Number of antidepressant prescriptions in primary sample, UK Biobank (by drug class and diagnosis) 15

Supplementary table 5. Number of patients with at least one prescription for antidepressant drug classes in primary sample, UK Biobank (by diagnosis) 16

Supplementary table 6. Number of patients with depression and anxiety diagnoses on or before index SSRI exposure in primary sample, UK Biobank (by diagnosis) 17

Supplementary table 7. Summary for Generation Scotland replication sample 18

Supplementary table 8. Switching patterns in primary sample, UK Biobank 20

Supplementary table 9. Switching patterns in Generation Scotland replication sample 21

Supplementary table 10. Statistical tests for distributions of SSRI switcher / non-switcher status across assessment centres and genotyping batches 22

Supplementary table 11. Odds ratio for association testing in primary sample, UK Biobank 23

Supplementary table 12. Estimates for SNP-based heritability 25

Supplementary table 13. Top 40 hit SNPs with p < 1e-5 for SSRI switching (full sample) 26

Supplementary table 14. Top 40 hit SNPs with p < 1e-5 for SSRI switching (≥ 1 MDD diagnosis record) 28

Supplementary table 15. Top 40 hit SNPs with p < 1e-5 for SSRI switching (≥ 2 MDD diagnosis records) 30

Supplementary Figures **32**

Supplementary figure 1. Schematic diagram to illustrate (A) a switcher from paroxetine (SSRI) to clomipramine (TCA); (B) a non-switcher for fluoxetine (SSRI). 32

Supplementary figure 2. Sample sizes for SSRI switching, stratified by diagnosis criteria 33

Supplementary figure 3. Distribution of prescriptions in primary sample, UK Biobank 34

Supplementary figure 4. Distribution plot for time to switch for SSRI switchers in primary sample, UK Biobank 36

Supplementary figure 5. Distribution of index SSRI used and age at index date for SSRI prescription of primary sample, UK Biobank 37

Supplementary figure 6. Sex-specific descriptive analysis for primary sample, UK Biobank 38

Supplementary figure 7. Alluvial plot for switching patterns in Generation Scotland replication sample 40

Supplementary figure 8: Distribution of switch patterns across assessment centres........... 41

Supplementary figure 9. Relationship between switching patterns and SSRI index date percentiles in UKB 42

Supplementary figure 10. BMI distribution at assessment for primary sample, UK Biobank 43

Supplementary figure 11: Distribution of neuroticism scores at assessment for primary sample 44

Supplementary figure 12. Distribution of demographic variables across assessment centres 45

Supplementary figure 13: GCTB estimates on *Pi* (polygenicity) and *S* (negative selection) 46

Supplementary figure 14. Heritability distribution for GCTB per iteration in MCMC sampler 47

Supplementary figure 15. Manhattan plot for SSRI switching (full sample) 48

Supplementary figure 16: Manhattan plot for SSRI switching (≥ 1 depression diagnosis record) 49

Supplementary figure 17: Manhattan plot for SSRI switching (≥ 2 depression diagnosis records) 50

Supplementary figure 18. QQ plot for SSRI switching (full sample)................................ 51

Supplementary figure 19. QQ plot for SSRI switching (≥ 1 depression diagnosis record). 52

Supplementary figure 20. QQ plot for SSRI switching (≥ 2 depression diagnosis records)53

References **54**

Supplementary Methods

Primary Sample – UK Biobank (UKB)

Sample description

The UKB is a population-wide prospective cohort study consisting of 488,377 participants recruited from the United Kingdom (UK) (1). All participants provided consent upon assessment, and completed self-assessment questions on sociodemographic and lifestyle characteristics, as well as self-reported diagnoses information (1). They also provided blood and saliva samples for genome-wide genotyping (see below). Several waves of follow-up assessments were conducted after initial assessment, including physical and mental health measurements in the form of mental health questionnaire (2).

This project has received approval to be conducted using the UK Biobank resource, under the project application number 82087.

Genotyping

All participants in UKB underwent genome-wide genotyping by either the UKB Axiom or the UK BiLEVE Axiom arrays, identifying 805,426 single nucleotide polymorphisms (SNPs) for 488,377 UKB participants (1). SNPs present on both genotyping assays above underwent quality control (QC), filtering SNPs with genotype missing calls > 5%, minor allele frequencies (MAF) < 0.0001 and failing QCs in multiple batches (1). This resulted in 670,739 SNPs being imputed using SHAPEIT3 in 487,442 participants, after filtering for missing genotype calls and heterozygosity in samples (1). Haplotype imputation was performed using Haplotype Reference Consortium (HRC) (3) and the merged UK10K and 1000 Genomes Phase 3 reference panels (4). This eventually resulted in 93,095,623 autosomal SNPs for UKB participants passing quality control steps (1).

Further QC was performed on genotyped data provided by the UKB. Genotyped SNPs were filtered off with missing genotyping rates (GENO) > 0.02, Hardy-Weinberg Equilibrium (HWE) p-value < 10^{-8} and minor allele frequencies (MAF) < 0.01. Individuals were filtered for

abnormal heterozygosity (as defined during centralised quality control). Population structure was assessed using 4-means clustering in the first two principal components (PCs), and only participants of European ancestry were retained. Relatedness of individuals was handled by REGENIE in subsequent analyses (5). Imputed SNPs were further filtered with an imputation quality (INFO) score threshold of 0.4.

Primary care records

According to documentation from UKB, there is currently no national system for collecting primary care records (6). Therefore, at the current release, UKB liaised with data suppliers and other intermediaries to establish linkage to primary care data for ~230,000 (~ 45%) UKB participants, all of whom have provided written consent for linkage to their health-related records.

For primary care records, clinical events were coded by READ v2 and CTV-3 (6). Prescription records in the primary care setting were coded by READ v2, British National Formulary (BNF) and Dictionary of Medicines and Devices (dm+d) (6). Prescriptions are available from the 1990s to 2018, with the start and end dates dependent on the databases linked to specific regions of practice. Only the dates of prescription are available with no information on dispensing. Prescription codes for SSRIs were extracted from all available records. Further details of coding systems, including READ codes, BNF and dm+d codes are available in documentations from UKB (6) and previous work on treatment-resistant depression (7). Details of the data providers and the coding schema used are summarised below.

Country	GP Computer System Supplier	Approx no. of UK Biobank participants	Clinical coding classification	Prescription coding
Scotland [a]	EMIS [b] / Vision [c]	27,000	• Read v2	• Read v2<br>• British National Formulary (BNF)
Wales [d]	EMIS / Vision	21,000	• Read v2	• Read v2

England	TPP[e]	165,000	• Clinical Terms Version 3 (CTV3 or Read v3)	• BNF
	Vision	18,000	• Read v2	• Read v2<br>• Dictionary of<br>• Medicines and Devices (dm+d)

[a] UK Biobank has engaged Albasoft (http://www.albasoft.co.uk/) (a third party data processor) to obtain data from GP practices in Scotland.

[b] EMIS Health (https://www.emishealth.com/) is a computer system supplier to the NHS and provides the EMIS Web practice management system.

[c] Vision Health (https://www.visionhealth.co.uk/) (previously InPS) is a computer system supplier and provides the Vision practice management system.

[d] Data from Wales have been obtained via the SAIL Databank (https://saildatabank.com/) hosted by the University of Swansea.

[e] TPP (https://www.tpp-uk.com/) is a computer system supplier and provides the SystmOne practice management system.

Replication Sample – Generation Scotland

Sample description

Generation Scotland (GenScot) is a family-based longitudinal study consisting around 24,000 volunteers to study the genetic and demographic characteristics of non-communicable disorders in Scotland (8). Participants aged between 35 and 65 years (and at least one first-degree relative aged above 18) were selected from primary care centres in Scotland from 2006 to 2011, resulting in a cohort of 23,960 participants. Participants provided blood and saliva samples for processing, including genome-wide genotyping. Notably, participants consented to the use of their linked electronic health records for research purposes. Linked data available for the GenScot cohort includes community-dispensed medications (Prescribing Information System, provided by Public Health Scotland), GP records (provided by Albasoft), inpatient, outpatient and registry records (Scottish Morbidity Records, provided by Public Health Scotland), and national death records (provided by Public Health Scotland). A depression-

focussed follow-up study was conducted for between 2015 and 2017, consisting of remote mental health questionnaires (N = 9,618) and in-person clinic visits (N = 1,188) (9).

Polygenic scoring

Polygenic scores (PGS) for major depressive disorder (MDD), antidepressant non-remission and schizophrenia were computed using PRS-CS-auto (10) under the GenoPred pipeline (version 1, 7-Feb-2024) (11). The study characteristics and details of GWAS summary statistics are available in **Supplementary table 3**. All three GWAS (12–14) were performed by the Psychiatric Genomics Consortium, with no UKB samples included in the meta-analysis. The technical details for GenoPred (11) pipeline is available at: https://opain.github.io/GenoPred/pipeline_technical.html.

To handle relatedness issues in association testing between switching and PGS, only one of the two related pairs with kinship coefficient < 0.044 (third-degree relatives) was retained (See also **Methods** in main text). Kinship coefficients were estimated using the KING (15) software, as detailed in UKB documentation (1).

GWAS summary statistics underwent quality control as per documentation in GenoPred pipeline, filtering SNPs with duplicated rsids, INFO scores < 0.9, MAF < 0.01, and invalid p-values ($p < 0$ or $p > 1$). Variants in GWAS summary statistics were also matched to a reference panel consisting of HapMap3 variants (16) based on 1000 Genomes Phase 3 (1KG) and Human Genome Diversity Project (HGDP) samples. Strand-flipping was performed based on matching with this genotype reference, and SNPs with MAF differences < 0.2 between GWAS summary statistics and reference panel were removed.

PRS-CS is a polygenic scoring method that performs Bayesian shrinkage on the effect size distribution of variants using a continuous shrinkage prior (10). The "auto" framework under PRS-CS utilised a half-Cauchy prior on the phi (Φ) hyperparameter which controlled the degree of sparseness of the distribution of SNP effect sizes (10), such that Φ was estimated from target data, without the need for an external validation dataset. In GenoPred pipeline, PGS were

computed in UKB, and reference PGS were also computed under the same framework consisting of 1KG European samples (17). The PGS computed in target samples were then re-scaled such that the mean and standard deviations were comparable to the 1KG European reference, so that the distributions of PGS and the results were comparable across samples.

Genome-wide association studies (GWASs)

Two GWASs were performed on patients with at least one and two GP diagnoses for MDD respectively using REGENIE (5). REGENIE divides association testing into 2 steps to enhance computational efficiency, and is well-suited to conduct genetic analyses on UKB. In Step 1, ridge regression was fitted into genome-wide genotyped SNPs divided into consecutive blocks of 1000 SNPs. The blocks of predictors from ridge regression were combined by another logistic ridge regression, with shrinkage parameters cross-validated by the leave-one-chromosome-out (LOCO) approach. These LOCO predictors were then used as covariates in Step 2 of REGENIE. Before running step 1 of REGENIE, the genotyped SNPs were pruned by linkage disequilibrium (LD), using R^2 threshold of 0.9 within a window size of 1000 markers and a block size of 100 markers (5). In Step 2, association testing was performed on imputed SNPs genome-wide (filtered by INFO < 0.4) and switching status in both samples, adjusting for LOCO predictors and additional covariates (see below). In both steps of REGENIE, associations were further adjusted for genetic sex, index date of SSRI and first ten PCs for population stratification. Firth correction was used to correct for inflated SNP effect sizes and subsequent Type 1 error, which was evident when sample prevalence figures were low (5).

SNP-based heritability (h^2_{SNP}) estimation

h^2_{SNP} estimation was performed on individual-level genotype data of both samples using two different methods, including genome-wide complex trait analysis (GCTA) version 1.94.1 (18) and Genome-wide Complex Trait Bayesian (GCTB) (19). Due to a lack of information on population prevalence of switching from SSRIs, h^2_{SNP} estimates were reported on an observed scale.

GCTA estimates SNP-based heritability under the genomic relatedness–based restricted maximum likelihood (GREML) framework (18). Genomic relationship matrices (GRM) were

constructed using genotyped SNPs that passed QC steps (see **Genotyping**) and further adjusted for incomplete tagging of causal SNPs. Genetic variance is subsequently estimated by fitting effects of genotyped SNPs (as GRM) in mixed linear model by REML. Related subjects were removed from the GRM with a cut-off at 0.05, and adjusted for covariates including first ten PCs for population stratification, genetic sex and index date of SSRI.

We also used the GCTB Bayes S framework in h^2_{SNP} estimation under the default settings of GCTB (19,20), using 21000 iterations in Markov chain Monte Carlo (MCMC) with the first 1000 MCMC chains discarded / burnt in. GCTB framework allows simultaneous estimation SNP effects and genetic architecture characteristics, such as degrees of polygenicity (20). Therefore in GCTB, the degree of polygenicity (Pi) and negative selection (S) were also reported. By default, GCTB constrained h^2_{SNP} to between 0 and 1 when running iterations for h^2_{SNP} estimation. As a result, h^2_{SNP} may be skewed and not normally distributed if true h^2_{SNP} is close to zero. To support the use of posterior mean and standard errors in h^2_{SNP} estimation, we reported the distributions of h^2_{SNP} estimates across all iterations, and with 1000 burn-in iterations removed in **Supplementary figure 13** support the distributions are normally distributed. To ensure the robustness of our findings, we further reported the posterior mode and the 95% highest posterior density (HPD) intervals of h^2_{SNP} estimates using GCTB, computed using the R package "HDInterval" (21).

Supplementary tables

Supplementary table 1. READ v3 diagnosis codes used in this study

Diagnosis	READ v3 code list
**Depression**	XE1YC, XE1ZZ, E1120, E1123, XaX53, E1124, E112z, E1122, E1121, E1125, E1126, XE1Y0, XSGom, XSGon, XE1ZY, X761L, E1130, E1133, XaX54, E1134, E1132, E1131, E1135, E1136, XE1Y1, E113z, XE1Zf, Eu334, XE1Zd, XE1Ze, Eu331, Eu330, XE1Zc, E1137, E291., Eu33y, XE1Za, XSGol, Eu321, X00Sb, XSGok, Eu320, XaCIs, X00SU, E2112, XE1Zb, E2B.., E2B1., E290z, E11y2, XaY2C, X00SQ, E112., E113., E11z2, E135., E2003, E204., Eu322, Eu323, Eu324, Eu325, Eu326, Eu32y, Eu32z, Eu33., Eu332, Eu333, Eu33z, Eu34., Eu3y1, Eu412, X00SO, X00SR, X00SS, Xa0wV, XaB9J, XaCHr, XaCHs, XaCIu, XE1aY, XM1GC, XSEGJ, XE1Xy
**Bipolar disorders**	XE1Xz, E11y0, E1170, E1173, E1174, E117z, E1172, E1171, E1175, E1176, E117., E1100, E1103, E1104, E1102, E1101, E1105, E1106, E1110, E1113, E1114, E1112, E1111, E1115, E1116, E111., E111z, E11z1, E11y3, Eu30y, XE1ZX, E11yz, E11y., E1160, E1163, E1164, E116z, E1162, E1161, E1165, E1166, E116., XE1ZW, E110z, Eu301, XE1ZV, X00SL, E2113, X00SN, XaY1Y, X00SM, Eu31z, E1140, E1143, E1144, E114z, E1142, E1141, E1145, E1146, Eu317, E1150, E1153, E1154, E115z, E1152, E1151, E1155, E1156, Eu314, Eu316, Eu313, Eu311, Eu312, E114., Eu310, E115., E11y1, E11.., E110., E11y2, E11z., E11z0, E2111, Eu30., Eu300, Eu302, Eu30z, Eu31., Eu315, Eu31y
**Psychosis**	XE1Xw, XE1Xy, XE1ZQ, Xa0s9, XE1ZR, XE2b8, E1000, E1070, E03y3, E1030, XE1ZU, E1010, E1020, Eu203, E1001, E1071, E1031, E1011, E1021, E100z, E100., E10.., Eu20z, Eu2.., E10z., E1005, Eu20., E107z, E1075, E107., Eu25z, XE2uT, XE2un, Eu25., E106., E130., E134., X00S8, E103z, E1035, E103., E12z., E1y.., E10yz, XE1Xx, XE1ZM, Eu25y, E13yz, E13y., XE1ZT, XE1Y3, Eu23y, XE1Y5, XaX52, E1..., E101z, E1015, E101., E1002, E1072, E1032, XE1Y2, E1012, E1022, E10y1, E102z, E1025, E102., E13y1, E10y0, X00SC, Eu231, E131., E1003, E1073, E1033, E1013, E1023, E1004, E1074, E1034, E1014, E1024, XE1ZS, E104., E10y., E11zz, E121., E123., E13.., E13z., E1410, E1411, E141z, Eu0z., Eu200, Eu201, Eu202, Eu204, Eu205, Eu206, Eu20y, Eu220, Eu230, Eu232, Eu233, Eu23z, Eu252, Eu2y., Eu2z., X00S6, X761M, XE1aM, XE1aO
**Substance abuse**	XE1Xu, XE1YR, XE1YS, XE1YY, XE1YZ, XE1Ya, XE1ZH, XE1ZI, XE1ZJ, XE2um, XE2n9, E0112, E2400, E2310, Xa1bX, E022., E014., E2470, E247z, E2473, E2472, E2471, XE1YW, E02yz, E02y., E01yz, E01y., E240z, E2403, E25y0, E25yz, E25y3, E25y2, E25y1, XE1Yb, E2550, E255z, E2553, E2552, E2551, E255., E2590, E259z, E2593, E2592, E2591, E259., E2540, E254z, E2543, E2542, E2541, E2530, E253z, E2533, E2532, E2531, E2560, E256z, E2563, E2562, E2561, E256., E2520, E252z, E2523, E2522, E2521, E252., E2580, E258z, E2583, E2582, E2581, E2570, E257z,

	E2573, E2572, E2571, E2500, E250z, E2503, E2502, E2501, XE1YX, E2594, E25z., Eu184, Eu183, Eu18z, Eu187, Eu185, Eu18y, Eu181, Eu186, Eu180, Eu18., Eu174, Eu173, Eu17z, Eu177, Eu175, Eu17y, Eu171, Eu172, Eu176, Eu170, Eu134, Eu133, Eu13z, Eu137, Eu135, Eu13y, Eu131, Eu132, Eu136, Eu130, Eu13., Eu154, Eu153, Eu15z, Eu157, Eu155, Eu15y, Eu151, Eu156, Eu150, Eu15., Eu114, Eu11z, Eu117, Eu115, Eu11y, Eu111, Eu116, Eu110, Eu11., Eu164, Eu163, Eu16z, XE1ZK, Eu165, Eu16y, Eu161, Eu166, Eu160, Eu16., XaKC3, XaKC2, XaKC8, XaKC6, XaKC4, XaKC7, XaKC0, XaKC1, XaKC5, XaKBz, XaKBy, Eu144, Eu143, Eu14z, Eu147, Eu145, Eu14y, Eu141, Eu142, Eu146, Eu140, Eu14., Eu124, Eu123, Eu12z, Eu127, Eu125, Eu12y, Eu121, Eu122, Eu126, Eu120, Eu12., Eu104, Eu103, Eu10z, XE1ZG, XE1ZF, Eu10y, Eu101, XE1ZE, Eu106, Eu100, Eu10., Eu1.., Eu194, Eu193, Eu19z, Eu197, Eu195, Eu19y, Eu191, XE1ZL, Eu196, Eu190, Eu19., Eu17., E0111, E011., E2410, E241z, E2413, E2412, E2411, E2450, E245z, E2453, E2452, E2451, XE1YV, E2460, E246z, E2463, E2462, E2461, E246., E2402, E2312, E2302, E24A., E020., E02z., E02.., E02y4, E0210, E021., E021z, E0211, E02y3, E02y1, E02y0, E02y2, E24z., E24.., E010., E2401, E2311, E2301, E2480, E248z, E2483, E2482, E2481, E248., E249., E2490, E249z, E2493, E2492, E2491, E242., E242z, E2420, E2423, E2422, E2421, E231z, E2313, XE1YQ, E0120, XE1YT, E243z, E2430, E2433, E2432, E2431, E2440, E244z, E2443, E2442, E2441, XE1YU, E01z., E01.., E015., E01y0, XaLWu, E013., E23z., E011z, E2300, E2303, E230z, E230., E258., E0110, E012., E23.., E231., E240., E241., E243., E244., E245., E247., E250., E253., E254., E257., E25y., Eu102, Eu105, Eu107, Eu112, Eu113, Eu152, Eu162, Eu167, Eu182, Eu192, XE1b0, XE1b2, XE1b6
**Anxiety disorders**	X7627, XaIvf, Xa3WJ, E200., E2000, XE1Y7, E2002, E2004, E2005, E200z, XE1YA, E2020, E2021, E2022, E2023, E2024, E2025, E2026, E2027, E2028, E2029, X762G, Xa7lj, E202C, X762T, E202E, XE1YB, Eu40., X00SV, X00SW, X00SX, X00SY, Eu40y, Eu40z, Eu41., Eu410, Eu413, XE1Zj, Eu41z, 146G., E2001, E202., E202A, E202B, E202D, E202z, E28z., Eu400, Eu401, Eu402, Eu403, Eu411, Eu41y, Ua1qa, Ua1qc, Ua1qd, Ua1qe, Ua1qS, Ua1qs, Ua1qt, Ua1qU, Ua1qV, Ua1qW, Ua1qX, Ua1qY, X00Sr, X00Sy, X50G2, X50G3, X50G5, X50G6, X50GI, X761a, X761b, X761c, X761d, X761e, X761f, X761G, X761g, X761h, X761i, X761j, X761k, X761l, X761m, X761n, X761p, X761q, X761r, X761R, X761t, X761T, X761U, X761u, X761v, X761V, X761w, X761W, X761x, X761X, X761y, X761Y, X761z, X761Z, X7620, X7621, X7622, X7623, X7624, X7625, X7626, X7628, X7629, X762a, X762A, X762b, X762B, X762C, X762E, X762F, X762H, X762I, X762J, X762K, X762L, X762M, X762N, X762O, X762P, X762R, X762S, X762U, X762V, X762W, X762X, X762Y, X762Z, Xa00r, Xa00s, Xa1a8, Xa1Ev, Xa3Vk, Xa3Vl, Xa3WI, XaB96, XaEKL, XaK2c, XE1aW

Supplementary table 2. Antidepressant names and respective drug classes

Antidepressant name	Antidepressant drug Class
Sertraline	Selective serotonin reuptake inhibitors
Citalopram	Selective serotonin reuptake inhibitors
Fluoxetine	Selective serotonin reuptake inhibitors
Escitalopram	Selective serotonin reuptake inhibitors
Paroxetine	Selective serotonin reuptake inhibitors
Fluvoxamine	Selective serotonin reuptake inhibitors
Dapoxetine	Selective serotonin reuptake inhibitors
Moclobemide	Monoamine oxidase inhibitors
Phenelzine	Monoamine oxidase inhibitors
Tranylcypromine	Monoamine oxidase inhibitors
Isocarboxazid	Monoamine oxidase inhibitors
Tranylcypromine_trifluoperazine	Combination
Bupropion	Norepinephrine and dopamine reuptake inhibitors
Reboxetine	Norepinephrine reuptake inhibitors
Viloxazine	Norepinephrine reuptake inhibitors
Trazodone	Serotonin antagonist and reuptake inhibitors
Nefazodone	Serotonin antagonist and reuptake inhibitors
Venlafaxine	Serotonin-norepinephrine reuptake inhibitors
Duloxetine	Serotonin-norepinephrine reuptake inhibitors
Mirtazapine	Other antidepressants
Vortioxetine	Other antidepressants
Agomelatine	Other antidepressants
Flupentixol	Phenothiazine antipsychotics
Mianserin	Tetracyclic antidepressants

Maprotiline	Tetracyclic antidepressants
Amitriptyline	Tricyclic antidepressants
Imipramine	Tricyclic antidepressants
Dosulepin	Tricyclic antidepressants
Lofepramine	Tricyclic antidepressants
Nortriptyline	Tricyclic antidepressants
Clomipramine	Tricyclic antidepressants
Trimipramine	Tricyclic antidepressants
Doxepin	Tricyclic antidepressants
Amoxapine	Tricyclic antidepressants
Protriptyline	Tricyclic antidepressants
Amitriptyline_perphenazine	Combination
Nortriptyline_fluphenazine	Combination

Caption

Antidepressant names are active ingredients listed as strings in primary care records in UK Biobank.

Supplementary table 3. GWAS summary statistics used for polygenic scoring

Phenotype	Author (s)	Publication Year	Ancestry	Sample size	Number of SNPs
**Non-remission**	Pain et al. (12)	2022	European	5,151<br>Remission<br>1,852<br>Non-remission<br>3,299	9,612,897
**MDD**	Wray et al. (13)	2018	European	143,265<br>Cases 45,591<br>Controls 97,674	11,345,536
**Schizophrenia**	Trubetskoy et al. (14)	2022	European	130,644<br>Cases 53,386<br>Controls 77,258	7,659,767

Abbreviations

MDD = major depressive disorder.

Supplementary table 4. Number of antidepressant prescriptions in primary sample, UK Biobank (by drug class and diagnosis)

Sample	Full Sample (N = 71,153[1])	≥ 1 depression diagnosis record (N = 43,510[1])	≥ 2 depression diagnosis records (N = 29,687[1])
**Drug class**			
Combination	2 (1, 6)	3 (1, 6)	2 (1, 6)
MAOI	7 (2, 25)	6 (2, 25)	6 (2, 26)
NDRI	2 (1, 2)	1 (1, 2)	1 (1, 2)
NRI	3 (1, 10)	3 (1, 9)	4 (1, 9)
Other antidepressants	5 (1, 17)	4 (1, 16)	4 (1, 16)
SARI	4 (1, 16)	4 (1, 14)	4 (1, 15)
SNRI	8 (2, 30)	9 (2, 32)	10 (3, 34)
SSRI	18 (7, 47)	22 (9, 54)	25 (10, 60)
TCA	6 (2, 22)	6 (2, 22)	6 (2, 23)
Tetracyclics	3 (1, 9)	4 (2, 8)	4 (2, 8)

Caption

[1] Number of prescriptions: Median (IQR).

Abbreviations

IQR = interquartile range; MAOI = monoamine oxidase inhibitors; NDRI = norepinephrine-dopamine reuptake inhibitors; NRI = norepinephrine reuptake inhibitors; SARI = serotonin antagonist and reuptake inhibitors; SNRI = serotonin–norepinephrine reuptake inhibitors; SSRI = selective serotonin reuptake inhibitors; TCA = tricyclic antidepressants.

Supplementary table 5. Number of patients with at least one prescription for antidepressant drug classes in primary sample, UK Biobank (by diagnosis)

Sample	Full Sample (N = 38,813[1])	≥ 1 depression diagnosis record (N = 22,706[1])	≥ 2 depression diagnosis records (N = 14,781[1])
**Drug class**			
SSRI	38813 (100%)	22706 (100%)	14781 (100%)
TCA	18125 (46.7%)	11320 (49.9%)	7749 (52.4%)
Other antidepressants	5268 (13.6%)	3517 (15.5%)	2623 (17.7%)
SNRI	5139 (13.2%)	3471 (15.3%)	2624 (17.8%)
SARI	2285 (5.9%)	1498 (6.6%)	1126 (7.6%)
Combination	831 (2.1%)	552 (2.4%)	409 (2.8%)
NRI	284 (0.7%)	187 (0.8%)	160 (1.1%)
NDRI	189 (0.5%)	105 (0.5%)	85 (0.6%)
MAOI	165 (0.4%)	117 (0.5%)	99 (0.7%)
Tetracyclics	54 (0.1%)	37 (0.2%)	31 (0.2%)

Caption

[1] Number of patients: count (percentage).

Abbreviations

MAOI = monoamine oxidase inhibitors; NDRI = norepinephrine-dopamine reuptake inhibitors; NRI = norepinephrine reuptake inhibitors; SARI = serotonin antagonist and reuptake inhibitors; SNRI = serotonin–norepinephrine reuptake inhibitors; SSRI = selective serotonin reuptake inhibitors; TCA = tricyclic antidepressants.

Supplementary table 6. Number of patients with depression and anxiety diagnoses on or before index SSRI exposure in primary sample, UK Biobank (by diagnosis)

Sample	Sample size	Proportion of patients with GP diagnosis on or before SSRI exposure	
		Depression	Depression or anxiety
≥ 1 depression diagnosis record	22706	16962 (74.7%) [a]	17525 (77.2%)
≥ 2 depression diagnoses records	14781	11502 (77.8%)	11902 (80.5%)

[a] Dates of depression diagnosis was not available for 39 participants in this sample.

Abbreviations

SSRI = selective serotonin reuptake inhibitors.

Supplementary table 7. Summary for Generation Scotland replication sample

Characteristic	Non-switchers (N = 1,279[1])	Switchers (N = 498[1])	p-value[2]
**Index SSRI**			0.017
Fluoxetine	433 (34%)	165 (33%)	
Citalopram	604 (47%)	190 (38%)	
Sertraline	199 (16%)	125 (25%)	
Escitalopram	19 (1.5%)	11 (2.2%)	
Paroxetine	24 (1.9%)	7 (1.4%)	
**Age on index date**	47 (36, 56)	51 (39, 62)	<0.001
**Unknown**	9	0	
**Time to first switch**	-	31 (31, 61)	
**Sex**			0.10
Female	989 (77%)	366 (73%)	
Male	290 (23%)	132 (27%)	
**Ethnic background**			0.019
**White**	1,179 (98%)	450 (97%)	
**Asian**	1 (<0.1%)	5 (1.1%)	
**Mixed**	3 (0.3%)	3 (0.6%)	
**Other**	2 (0.2%)	0 (0%)	
**Not specified**	13 (1.1%)	8 (1.7%)	
**Unknown**	81	32	
**Body mass index**	26.2 (23.1, 30.2)	25.8 (22.3, 29.5)	0.057
Unknown	68	31	
**Neuroticism score**	6 (4, 9)	6 (3, 9)	0.10
Unknown	173	60	
**Family history of depression**	335 (27%)	123 (26%)	0.7
Unknown	32	19	
**Qualifications**			0.034
None of the above	76 (6.7%)	37 (8.9%)	
Secondary	264 (23%)	116 (28%)	
Vocational	253 (22%)	93 (22%)	
Further	134 (12%)	44 (11%)	
University Degree	331 (29%)	91 (22%)	
Not available	81 (7.1%)	36 (8.6%)	
**Unknown**	140	81	
**Annual income**			0.7
Less than 10,000	121 (11%)	55 (14%)	
10,000-30,000	374 (34%)	141 (35%)	
30,000-50,000	280 (25%)	106 (26%)	
50,000-70,000	164 (15%)	41 (10%)	
Greater than 70,000	81 (7.4%)	21 (5.2%)	
Not available	82 (7.4%)	42 (10%)	
**Scottish Index of Multiple Deprivation Quintile**			0.5
**1**	209 (17%)	93 (20%)	

**2**	200 (17%)	81 (17%)
**3**	213 (18%)	75 (16%)
**4**	281 (23%)	96 (21%)
**5**	304 (25%)	122 (26%)
**Unknown**	72	31

Caption

[1] n (%); Median (IQR).

[2] Kruskal-Wallis rank sum test; Wilcoxon rank sum test; Pearson’s Chi-squared test.

Abbreviations

SSRI = selective serotonin reuptake inhibitors

Supplementary table 8. Switching patterns in primary sample, UK Biobank

SSRI (pre-switch)		Fluoxetine (N = 1,687[1])	Paroxetine (N = 621[1])	Citalopram (N = 1,586[1])	Sertraline (N = 1,024[1])	Escitalopram (N = 201[1])	Fluvoxamine (N = 14[1])	Overall (N = 5133[1])
**Drug class (post-switch)**	**TCA**	572 (34%)	248 (40%)	467 (29%)	254 (25%)	49 (24%)	7 (50%)	**1597 (31%)**
	**NRI**	3 (0.2%)	4 (0.6%)	4 (0.3%)	2 (0.2%)	0 (0%)	0 (0%)	**13 (0.3%)**
	**Another SSRI**	785 (47%)	273 (44%)	737 (46%)	478 (47%)	102 (51%)	5 (36%)	**2380 (46%)**
	**Combination**	14 (0.8%)	14 (2.3%)	16 (1.0%)	7 (0.7%)	4 (2.0%)	0 (0%)	**55 (1.1%)**
	**Other antidepressants**	131 (7.8%)	14 (2.3%)	199 (13%)	172 (17%)	17 (8.5%)	1 (7.1%)	**534 (10%)**
	**SNRI**	118 (7.0%)	49 (7.9%)	111 (7.0%)	79 (7.7%)	24 (12%)	0 (0%)	**381 (7.4%)**
	**SARI**	62 (3.7%)	15 (2.4%)	50 (3.2%)	30 (2.9%)	5 (2.5%)	1 (7.1%)	**163 (3.2%)**
	**MAOI**	1 (<0.1%)	2 (0.3%)	2 (0.1%)	2 (0.2%)	0 (0%)	0 (0%)	**7 (0.1%)**
	**NDRI**	0 (0%)	2 (0.3%)	0 (0%)	0 (0%)	0 (0%)	0 (0%)	**2 (< 0.1%)**
	**Tetracyclics**	1 (<0.1%)	0 (0%)	0 (0%)	0 (0%)	0 (0%)	0 (0%)	**1 (<0.1%)**

Caption

[1] Number of patients with SSRI pre-switching: count (percentage).

Abbreviations

MAOI = monoamine oxidase inhibitors; NDRI = norepinephrine-dopamine reuptake inhibitors; NRI = norepinephrine reuptake inhibitors; SARI = serotonin antagonist and reuptake inhibitors; SNRI = serotonin–norepinephrine reuptake inhibitors; SSRI = selective serotonin reuptake inhibitors; TCA = tricyclic antidepressants.

Supplementary table 9. Switching patterns in Generation Scotland replication sample

	SSRI (pre-switch)	Fluoxetine (N = 165[1])	Paroxetine (N = 7[1])	Citalopram (N = 190[1])	Sertraline (N = 125[1])	Escitalopram (N = 11[1])	Fluvoxamine 0 (0%)	Overall (N = 498[1])
**Drug class (post-switch)**	**TCA**	26 (15.8%)	0 (0%)	30 (15.8%)	16 (12.8%)	1 (9.1%)	0 (0%)	**73 (14.7%)**
	**NRI**	0 (0%)	0 (0%)	0 (0%)	0 (0%)	0 (0%)	0 (0%)	**0 (0%)**
	**Another SSRI**	81 (49.1%)	2 (28.6%)	99 (52.1%)	48 (38.4%)	7 (63.6%)	0 (0%)	**237 (47.6%)**
	**Combination**	0 (0%)	0 (0%)	0 (0%)	0 (0%)	0 (0%)	0 (0%)	**0 (0%)**
	**Other antidepressants**	37 (22.4%)	2 (28.6%)	42 (22.1%)	45 (36.0%)	2 (18.2%)	0 (0%)	**128 (25.7%)**
	**SNRI**	17 (10.3%)	3 (42.8%)	16 (8.4%)	13 (10.4%)	1 (9.1%)	0 (0%)	**50 (10.0%)**
	**SARI**	4 (2.4%)	0 (0%)	3 (1.6%)	2 (1.6%)	0 (0%)	0 (0%)	**9 (1.8%)**
	**MAOI**	0 (0%)	0 (0%)	0 (0%)	1 (0.8%)	0 (0%)	0 (0%)	**1 (0.2%)**
	**NDRI**	0 (0%)	0 (0%)	0 (0%)	0 (0%)	0 (0%)	0 (0%)	**0 (0%)**
	**Tetracyclics**	0 (0%)	0 (0%)	0 (0%)	0 (0%)	0 (0%)	0 (0%)	**0 (0%)**

Caption

[1] Number of patients with SSRI pre-switching: count (percentage).

Abbreviations

MAOI = monoamine oxidase inhibitors; NDRI = norepinephrine-dopamine reuptake inhibitors; NRI = norepinephrine reuptake inhibitors; SARI = serotonin antagonist and reuptake inhibitors; SNRI = serotonin–norepinephrine reuptake inhibitors; SSRI = selective serotonin reuptake inhibitors; TCA = tricyclic antidepressants.

Supplementary table 10. Statistical tests for distributions of SSRI switcher / non-switcher status across assessment centres and genotyping batches

Sample	Covariate	df	p-value
**Full sample**	Genotype batch	105	0.346067
	Assessment centre	21	**2.54E-08***
**≥ 1 depression diagnosis record**	Genotype batch	105	0.117948
	Assessment centre	20	**7.92E-06***
**≥ 2 MDD diagnosis records**	Genotype batch	105	0.292841
	Assessment centre	20	**0.024137***

Caption

Statistical significance assessed by Kruskal-Wallis test.

* p-value < 0.05.

Supplementary table 11. Odds ratio for association testing in primary sample, UK Biobank

Study outcome		All sample		≥ 1 depression diagnosis record		≥ 2 depression diagnosis records	
		OR (95% CI)[1]	p[2]	OR (95% CI)[1]	p[2]	OR (95% CI)[1]	p[2]
Educational levels	None of the above	Reference					
	Secondary	0.82 [0.75-0.89]		0.87 [0.78-0.97]		0.88 [0.77-1.00]	
	Vocational	0.86 [0.77-0.95]		0.89 [0.78-1.02]		0.88 [0.74-1.04]	
	Further	0.78 [0.70-0.87]		0.86 [0.74-0.99]		0.77 [0.65-0.92]	
	University Degree	0.73 [0.67-0.79]	**1.53E-10****	0.78 [0.69-0.87]	**0.001****	0.79 [0.69-0.91]	**0.013***
Annual income	Less than £18,000	Reference					
	£18,000-£31,000	0.87 [0.80-0.95]		0.89 [0.80-0.99]		0.88 [0.77-1.01]	
	£31,000-£52,000	0.77 [0.71-0.84]		0.8 [0.71-0.89]		0.78 [0.68-0.90]	
	£52,000-£100,000	0.7 [0.62-0.77]		0.73 [0.63-0.83]		0.72 [0.61-0.85]	
	Greater than £100,000	0.66 [0.53-0.83]	**6.79E-15****	0.61 [0.44-0.82]	**4.92E-07****	0.75 [0.52-1.06]	**1.43E-04****
Townsend Deprivation Index		1.01 [1.00-1.02]	0.085	1.01 [0.99-1.02]	0.268	1.00 [0.99-1.02]	0.722
Family history[3]		1.08 [1.00-1.16]	**0.048***	1.09 [0.99-1.19]	0.084	1.10 [0.99-1.23]	0.088
PGS_{MDD}		1.02 [0.99-1.06]	0.138	1.00 [0.96-1.05]	0.848	0.99 [0.94-1.04]	0.73
$PGS_{non\text{-}rem}$		1.03 [1.00-1.07]	**0.029***	1.04 [1.00-1.08]	**0.031***	1.07 [1.02-1.12]	**0.007****
PGS_{SCZ}		1.02 [0.99-1.05]	0.212	1.00 [0.96-1.04]	0.903	0.99 [0.95-1.04]	0.737

Abbreviations

MDD = major depressive disorder; non-rem = non-remission from antidepressants; OR = odds ratio; PGS = polygenic score; SCZ = schizophrenia; SSRI = selective serotonin reuptake inhibitors.

[1] Associations controlled for index date of SSRI, sex, assessment centre and 10 principal components for population stratification.

[2] p-values tested by likelihood ratio test.

[3] Family history of severe depression.

* $p < 0.05$.

** $p < 0.00714$ (Bonferroni correction $n = 7$).

Supplementary table 12. Estimates for SNP-based heritability

Sample	Method	$h^2_{observed}$	SE	p-value
**Full sample**	**GCTA**	**0.0268**	**0.0158**	**0.038***
	**GCTB**	**0.0242**	**0.0095**	**0.005****
**≥ 1 depression diagnosis record**	**GCTA**	**0.0431**	**0.0268**	**0.048***
	**GCTB**	**0.0398**	**0.016**	**0.005****
**≥ 2 depression diagnosis records**	**GCTA**	0.0216	0.0394	0.293
	**GCTB**	0.035	0.025	0.08

* p-value < 0.05.

** p-value < 0.01.

Abbreviations

GCTA = genome-wide complex trait analysis; GCTB = Genome-wide Complex Trait Bayesian; $h^2_{observed}$ = SNP-based heritability (on observed scale); LDSC = linkage disequilibrium score regression; SE = standard error.

Supplementary table 13. Top 40 hit SNPs with p < 1e-5 for SSRI switching (full sample)

rsid	chr	pos	EA	MAF	BETA	SE	p	Nearest Gene	dist	func	CADD
rs56206563	8	33851064	T	0.0159	0.46512	0.0864874	2.53E-07	RP11-317N12.1:RP1-273G13.3	00:00	ncRNA_intronic	1.729
rs72630918	8	33659446	G	0.0338	0.377248	0.0735669	7.23E-07	RP11-317N12.1	0	ncRNA_intronic	1.333
rs1127390	16	81010073	T	0.2296	-0.133574	0.0273119	8.20E-07	CMC2	0	exonic	0.248
rs9931292	16	81009568	G	0.2296	-0.133457	0.0273087	8.36E-07	CMC2	129	downstream	1.266
rs10110388	8	33569261	T	0.02286	-0.424367	0.0830298	8.78E-07	RP11-317N12.1	0	ncRNA_intronic	0.194
rs72630978	8	33786522	A	0.0159	0.455454	0.0889054	8.90E-07	RP11-317N12.1	0	ncRNA_intronic	2.78
rs9921448	16	81009490	G	0.2296	-0.133087	0.0273102	8.98E-07	CMC2	207	downstream	0.686
rs2028594	8	33564880	G	0.02187	-0.428417	0.0841754	9.95E-07	RP11-317N12.1	0	ncRNA_intronic	0.097
rs2549855	16	81026852	G	0.2326	0.132147	0.0272665	1.03E-06	CMC2	0	intronic	0.96
rs11378897	8	33924941	TA	0.0159	-0.46628	0.0916406	1.04E-06	RP11-317N12.1	28414	intergenic	5.422
rs2123627	16	81021816	A	0.2336	0.13206	0.027274	1.06E-06	CMC2	0	intronic	1.728
rs874054	16	81006142	C	0.2296	-0.1319	0.0272964	1.11E-06	CMC2	3555	intergenic	3.58
rs874053	16	81006107	G	0.2296	-0.131886	0.0272961	1.11E-06	CMC2	3590	intergenic	1.322
rs61846889	10	50970429	A	0.1252	0.159793	0.0323949	1.12E-06	OGDHL	3	upstream	20.5
rs56297803	16	81006252	AT	0.2296	-0.131617	0.0273023	1.18E-06	CMC2	3445	intergenic	0.336
rs2911149	16	81026309	A	0.2336	0.131376	0.0272683	1.20E-06	CMC2	0	intronic	0.352
rs2602446	16	81017915	C	0.2316	0.131524	0.0273056	1.20E-06	CMC2	0	intronic	4.031
rs2549858	16	81026994	A	0.2306	0.131228	0.027258	1.22E-06	CMC2	0	intronic	2.822
rs2549859	16	81027003	G	0.2306	0.131228	0.027258	1.22E-06	CMC2	0	intronic	4.164
rs2549856	16	81026927	T	0.2326	0.131216	0.0272585	1.22E-06	CMC2	0	intronic	1.951
rs2549857	16	81026958	A	0.2326	0.131216	0.0272585	1.22E-06	CMC2	0	intronic	0.139
rs34844332	16	81009520	AT	0.2316	-0.132042	0.0274481	1.24E-06	CMC2	177	downstream	0.127
rs2167890	16	81021920	C	0.1769	0.144854	0.0302595	1.31E-06	CMC2	0	intronic	0.162
rs2549825	16	81018159	T	0.1769	0.144631	0.0302891	1.39E-06	CMC2	0	intronic	0.302
rs2167889	16	81021472	A	0.2316	0.130433	0.0272486	1.40E-06	CMC2	0	intronic	3.353
16:81023707_CT_C	16	81023707	C	0.2326	0.130374	0.0272401	1.41E-06	CMC2	0	intronic	0.564
rs35195025	16	81023304	TATC	0.2316	0.130322	0.0272501	1.43E-06	CMC2	0	intronic	7.19
rs2549839	16	81023235	T	0.2316	0.1302	0.0272425	1.46E-06	CMC2	0	intronic	0.783
rs2549844	16	81023861	C	0.2316	0.130012	0.0272089	1.47E-06	CMC2	0	intronic	5.334
rs2549822	16	81015560	T	0.1769	0.144057	0.0302687	1.51E-06	CMC2	0	intronic	3.954
rs1477381	16	81014700	C	0.2296	0.130154	0.0273036	1.55E-06	CMC2	0	intronic	6.362
rs2549834	16	81022544	T	0.1769	0.143713	0.0302653	1.59E-06	CMC2	0	intronic	1.066
rs2911148	16	81021894	G	0.2316	0.129669	0.027242	1.61E-06	CMC2	0	intronic	0.584
rs2549833	16	81022232	C	0.2316	0.129652	0.0272409	1.61E-06	CMC2	0	intronic	1.661

rs2549832	16	81020141	G	0.1769	0.14356	0.0302571	1.62E-06	CMC2	0	intronic	0.735
rs2602433	16	81029003	A	0.2336	0.129635	0.0272479	1.63E-06	CMC2	0	intronic	2.795
rs2549838	16	81023210	T	0.2316	0.129549	0.0272385	1.64E-06	CMC2	0	intronic	4.09
rs2549836	16	81023081	C	0.2316	0.129551	0.0272389	1.64E-06	CMC2	0	intronic	2.37
rs2549837	16	81023115	A	0.2316	0.129543	0.0272387	1.64E-06	CMC2	0	intronic	11.46
rs2316923	16	81024821	G	0.2316	0.129442	0.0272403	1.67E-06	CMC2	0	intronic	0.74

Caption

Annotation of variants was performed by FUMA tool (https://fuma.ctglab.nl/).

Abbreviations

chr = chromosome; dist = distance to the nearest gene; EA = effect allele; func = functions annotated from ANNOVAR; MAF = minor allele frequency; MDD = major depressive disorder; pos = position on hg19; rsid = variant ID; SE = standard error; SSRI = selective serotonin reuptake inhibitors.

Supplementary table 14. Top 40 hit SNPs with p < 1e-5 for SSRI switching (≥ 1 MDD diagnosis record)

rsid	chr	pos	EA	MAF	BETA	SE	p	Nearest Gene	dist	func	CADD
rs113737796	14	60845897	C	0.03877	0.351487	0.068506	6.78E-07	C14orf39	17289	intergenic	0.906
rs17097347	14	60817408	T	0.03777	0.351048	0.068594	7.20E-07	CTD-2568P8.1	26782	intergenic	0.906
14:60854762_TA_T	14	60854762	T	0.03777	0.348426	0.068639	8.80E-07	C14orf39	8424	intergenic	5.085
rs191325339	14	60889751	C	0.03777	0.343496	0.068264	1.09E-06	C14orf39	0	intronic	0.896
rs17097534	14	60961735	T	0.03777	0.345691	0.068743	1.11E-06	C14orf39:SALL4P7	00:00	ncRNA_exonic	8.925
14:60901561_GTTATA_G	14	60901561	G	0.03777	0.342844	0.069111	1.53E-06	C14orf39	0	intronic	0.735
rs75823917	14	60981509	A	0.03777	0.338341	0.068936	1.96E-06	C14orf39	0	intronic	3.913
rs12273649	11	24509974	T	0.06262	0.270512	0.055663	2.06E-06	LUZP2	8541	intergenic	0.705
rs149792878	14	49350551	T	0.0159	-0.58276	0.131428	2.07E-06	SNORD112	60098	intergenic	0.179
rs77206066	17	7959125	C	0.04374	0.330666	0.067733	2.19E-06	RP11-599B13.3	417	downstream	5.157
rs79837728	14	60998509	G	0.03777	0.338002	0.06923	2.21E-06	RP11-1042B17.3	0	ncRNA_intronic	9.689
rs116052952	2	2.41E+08	C	0.02286	0.462469	0.094058	2.36E-06	AC093802.1	28247	intergenic	5.749
14:60900965_TATA_T	14	60900965	T	0.03181	0.366986	0.075345	2.44E-06	C14orf39	0	intronic	0.775
rs2125950	8	2928234	G	0.4036	-0.14009	0.029636	2.48E-06	CSMD1	0	intronic	3.327
rs61949308	13	47238572	C	0.02187	-0.54658	0.124029	2.66E-06	LRCH1	0	intronic	0.007
rs2125951	8	2928227	G	0.4036	-0.13937	0.029636	2.79E-06	CSMD1	0	intronic	2.344
rs11878951	19	48792490	C	0.174	0.176505	0.037358	3.05E-06	ZNF114	1624	intergenic	3.59
rs140183378	4	43354672	A	0.02783	0.398119	0.08258	3.30E-06	RP11-1E6.1	7054	intergenic	0.55
rs202177809	4	43269936	GC	0.02783	0.4279	0.088743	3.35E-06	RP11-395F4.1	32962	intergenic	0.994
rs146536531	4	43358827	A	0.02783	0.396469	0.082554	3.59E-06	RP11-1E6.1	11209	intergenic	0.97
rs147130265	4	43357778	T	0.02783	0.395476	0.082565	3.80E-06	RP11-1E6.1	10160	intergenic	2.617
rs9651583	11	24507798	C	0.06262	0.261887	0.05543	3.87E-06	LUZP2	10717	intergenic	2.834
rs17457944	4	43356830	T	0.02783	0.395137	0.082567	3.87E-06	RP11-1E6.1	9212	intergenic	0.331
rs142825483	4	43356776	T	0.02783	0.395119	0.082567	3.87E-06	RP11-1E6.1	9158	intergenic	11.26
rs138323020	4	43356669	A	0.02783	0.395033	0.082569	3.90E-06	RP11-1E6.1	9051	intergenic	2.884
rs17536050	4	43355721	C	0.02783	0.394709	0.082568	3.96E-06	RP11-1E6.1	8103	intergenic	2.559
rs140355025	4	43355434	A	0.02783	0.394516	0.082571	4.01E-06	RP11-1E6.1	7816	intergenic	2.468
rs75824620	4	43359332	A	0.02783	0.395775	0.082849	4.02E-06	RP11-1E6.1	11714	intergenic	7.517
rs149873481	4	43354979	A	0.02783	0.39437	0.082571	4.04E-06	RP11-1E6.1	7361	intergenic	1.013
4:43358173_CTA_C	4	43358173	C	0.02783	0.395357	0.082788	4.04E-06	RP11-1E6.1	10555	intergenic	0.919
rs150723362	4	43354843	C	0.02783	0.39432	0.082572	4.05E-06	RP11-1E6.1	7225	intergenic	1.758
rs10456430	6	11763008	A	0.04274	-0.32428	0.072744	4.09E-06	ADTRP	0	intronic	4.281
rs146109701	4	43354470	T	0.02783	0.396238	0.083205	4.31E-06	RP11-1E6.1	6852	intergenic	0.172
rs142999246	4	43354458	T	0.02783	0.396196	0.083207	4.32E-06	RP11-1E6.1	6840	intergenic	0.146

rs189742614	4	43354459	G	0.02783	0.396196	0.083207	4.32E-06	RP11-1E6.1	6841	intergenic	1.258
rs140176922	4	43352617	C	0.02783	0.392782	0.082552	4.38E-06	RP11-1E6.1	4999	intergenic	1.201
rs144747495	4	43354012	G	0.02783	0.392761	0.082558	4.39E-06	RP11-1E6.1	6394	intergenic	1.448
rs138315122	4	43354240	C	0.02783	0.392754	0.08256	4.40E-06	RP11-1E6.1	6622	intergenic	5.636
rs138685627	4	43353958	G	0.02783	0.392719	0.082559	4.41E-06	RP11-1E6.1	6340	intergenic	0.817
rs150954018	4	43353407	A	0.02783	0.392551	0.082558	4.45E-06	RP11-1E6.1	5789	intergenic	0.521

Caption

Annotation of variants was performed by FUMA tool (https://fuma.ctglab.nl/).

Abbreviations

chr = chromosome; dist = distance to the nearest gene; EA = effect allele; func = functions annotated from ANNOVAR; MAF = minor allele frequency; MDD = major depressive disorder; pos = position on hg19; rsid = variant ID; SE = standard error; SSRI = selective serotonin reuptake inhibitors.

Supplementary table 15. Top 40 hit SNPs with p < 1e-5 for SSRI switching (≥ 2 MDD diagnosis records)

rsid	chr	pos	EA	MAF	BETA	SE	p	Nearest Gene	dist	func	CADD
rs7669265	4	76612386	G	0.04771	-0.468464	0.084515	9.05E-08	G3BP2	0	intronic	1.906
rs6531785	4	76557406	G	0.04374	-0.478522	0.0864935	9.64E-08	CDKL2	1505	intergenic	5.689
rs12518815	5	25130699	A	0.3907	-0.186432	0.037506	5.99E-07	RP11-549K20.1	0	ncRNA_intronic	1.255
rs114149662	1	192934797	C	0.01392	0.692656	0.134315	9.50E-07	LINC01032	17409	intergenic	0.95
rs116052952	2	240752825	C	0.02286	0.564921	0.111459	1.22E-06	AC093802.1	28247	intergenic	5.749
rs75382569	10	8052328	G	0.09046	0.295131	0.0601542	1.67E-06	TAF3	0	intronic	0.371
rs145453945	6	116666178	A	0.1342	0.225461	0.0466309	1.94E-06	DSE:RP1-93H18.1	00:00	ncRNA_intronic	0.735
rs11753710	6	116671302	G	0.1342	0.225467	0.0466323	1.94E-06	DSE:RP1-93H18.1	00:00	ncRNA_intronic	0.424
rs113698755	6	116678687	TC	0.1352	0.225673	0.0468486	2.11E-06	DSE:RP1-93H18.1	00:00	ncRNA_intronic	0.975
rs112711055	15	68339776	G	0.04175	-0.551114	0.123753	2.27E-06	PIAS1	6740	intergenic	7.168
rs149792878	14	49350551	T	0.0159	-0.726296	0.16712	2.32E-06	SNORD112	60098	intergenic	0.179
rs145987863	1	192964414	C	0.01392	0.658682	0.132881	2.35E-06	ZNF101P2	606	upstream	7.748
6:116672075_GA_G	6	116672075	G	0.1342	0.224654	0.0470485	2.58E-06	DSE:RP1-93H18.1	00:00	ncRNA_intronic	3.445
rs17077864	6	116672939	G	0.1342	0.222368	0.0467968	2.88E-06	DSE:RP1-93H18.1	00:00	ncRNA_intronic	4.704
rs141713005	1	193002886	A	0.01292	0.676011	0.137814	2.99E-06	UCHL5	0	intronic	4.447
rs73548973	6	116651294	C	0.1342	0.219056	0.0463135	3.17E-06	DSE:RP1-93H18.1	00:00	ncRNA_intronic	4.464
rs79325251	12	15197630	C	0.0328	0.393047	0.0819221	3.41E-06	RP11-508P1.2	38012	intergenic	0.824
rs141588653	12	15213368	A	0.03181	0.392222	0.0818963	3.55E-06	RERG	47348	intergenic	1.662
rs2089518	5	25358348	T	0.2992	0.185837	0.0404418	3.68E-06	RP11-184E9.2	36892	intergenic	0.287
rs59504977	6	116674329	C	0.1352	0.219914	0.0468233	3.73E-06	DSE:RP1-93H18.1	00:00	ncRNA_intronic	16.16
rs112851235	13	21567608	T	0.01292	-1.08379	0.268922	4.17E-06	LATS2	0	intronic	0.71
rs146584554	12	15177708	A	0.03479	0.387927	0.0817136	4.34E-06	RP11-508P1.2	18090	intergenic	0.579
rs7738448	6	116676685	A	0.1352	0.218288	0.0468421	4.43E-06	DSE:RP1-93H18.1	00:00	ncRNA_intronic	0.038
rs59149647	6	116679029	A	0.1352	0.218262	0.0468439	4.45E-06	DSE:RP1-93H18.1	00:00	ncRNA_intronic	0.124
rs113865485	9	116229738	A	0.02087	0.461864	0.0968897	4.45E-06	RGS3	0	intronic	0.127
rs60749937	6	116648592	A	0.1332	0.215926	0.0463699	4.48E-06	DSE:RP1-93H18.1	00:00	ncRNA_intronic	9.537
rs57818125	6	116648060	T	0.1332	0.215863	0.0463715	4.51E-06	DSE:RP1-93H18.1	00:00	ncRNA_intronic	9.135
rs8040839	15	44118890	T	0.09543	0.257496	0.0550392	4.59E-06	WDR76	270	upstream	3.95
rs1160705	5	25128668	T	0.4324	-0.165325	0.0363929	5.32E-06	RP11-549K20.1	0	ncRNA_intronic	5.354
rs116260600	2	110006345	A	0.0159	-0.924414	0.227852	5.33E-06	SH3RF3	0	intronic	1.75
rs111294065	15	68575696	C	0.03777	-0.559589	0.130372	5.33E-06	FEM1B	0	intronic	4.238
rs147678158	2	212104028	G	0.01093	0.738721	0.154078	5.47E-06	AC013404.1	59637	intergenic	1.812
rs112889512	9	116194189	T	0.0328	0.418393	0.0889459	5.55E-06	C9orf43	2224	intergenic	1.134
rs2125950	8	2928234	G	0.4036	-0.165104	0.0362821	5.84E-06	CSMD1	0	intronic	3.327

rs162253	17	3295368	G	0.4225	0.163402	0.0360851	5.87E-06	OR1E1	5029	intergenic	2.571
rs73548954	6	116644488	T	0.1471	0.211545	0.0460265	5.87E-06	DSE:RP1-93H18.1	00:00	ncRNA_intronic	4.493
rs36019747	1	186463784	G	0.01988	-0.546777	0.128017	5.88E-06	GS1-304P7.3	27120	intergenic	2.406
rs11113754	12	108525445	G	0.2217	0.190136	0.0415799	6.08E-06	WSCD2	0	intronic	3.834
rs11113755	12	108525533	T	0.2217	0.19014	0.0415821	6.08E-06	WSCD2	0	UTR5	12.56
rs11113756	12	108525595	G	0.2217	0.190143	0.0415831	6.08E-06	WSCD2	0	UTR5	6.547

Caption

Annotation of variants was performed by FUMA tool (https://fuma.ctglab.nl/).

Abbreviations

chr = chromosome; dist = distance to the nearest gene; EA = effect allele; func = functions annotated from ANNOVAR; MAF = minor allele frequency; MDD = major depressive disorder; pos = position on hg19; rsid = variant ID; SE = standard error; SSRI = selective serotonin reuptake inhibitors.

Supplementary Figures

Supplementary figure 1. Schematic diagram to illustrate (A) a switcher from paroxetine (SSRI) to clomipramine (TCA); (B) a non-switcher for fluoxetine (SSRI).

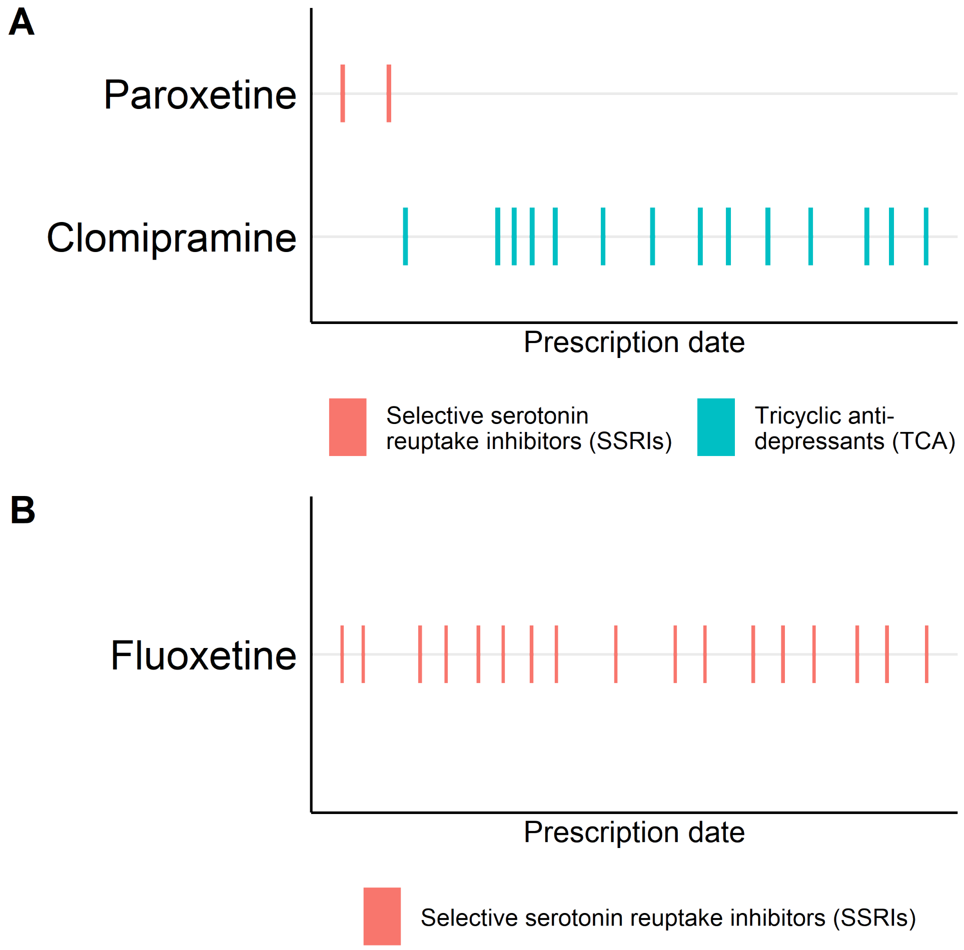

Abbreviations

SSRI = selective serotonin reuptake inhibitors; TCA = tricyclic antidepressants.

Supplementary figure 2. Sample sizes for SSRI switching, stratified by diagnosis criteria

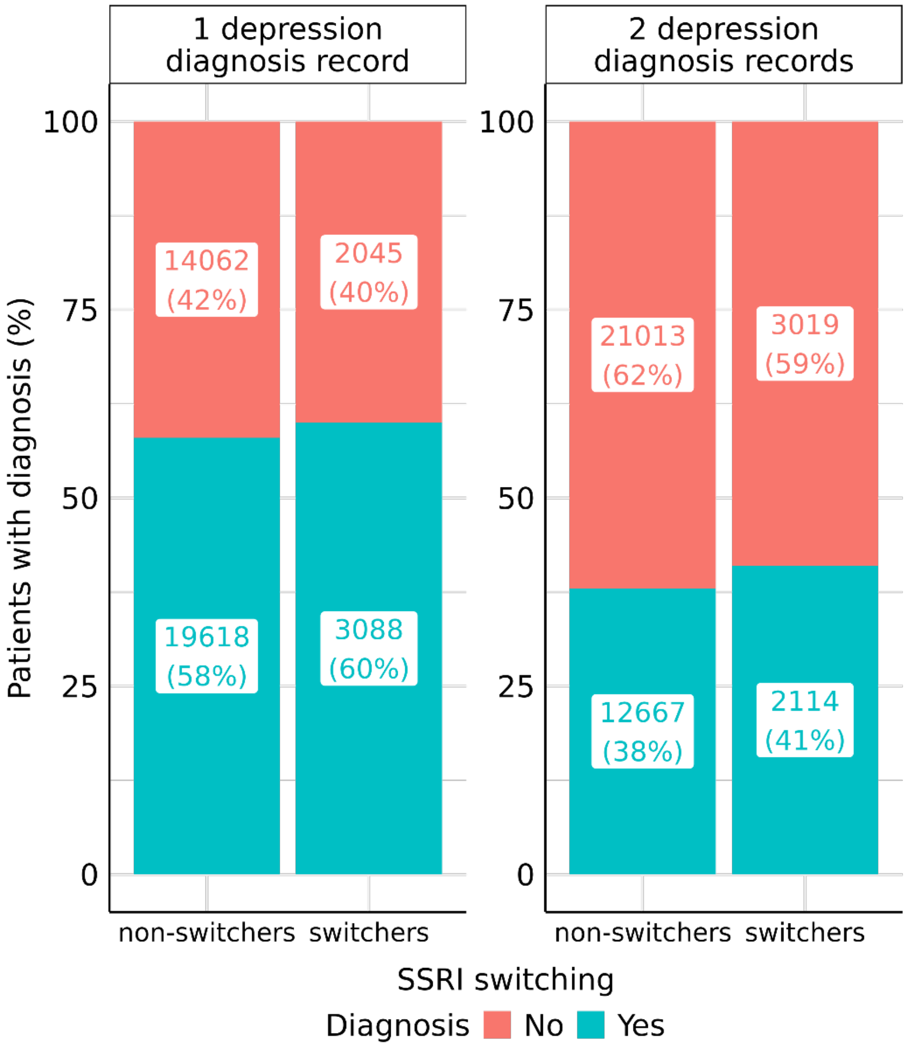

Caption

Numbers expressed as count (percentage).

Abbreviations

SSRI = selective serotonin reuptake inhibitors.

Supplementary figure 3. Distribution of prescriptions in primary sample, UK Biobank

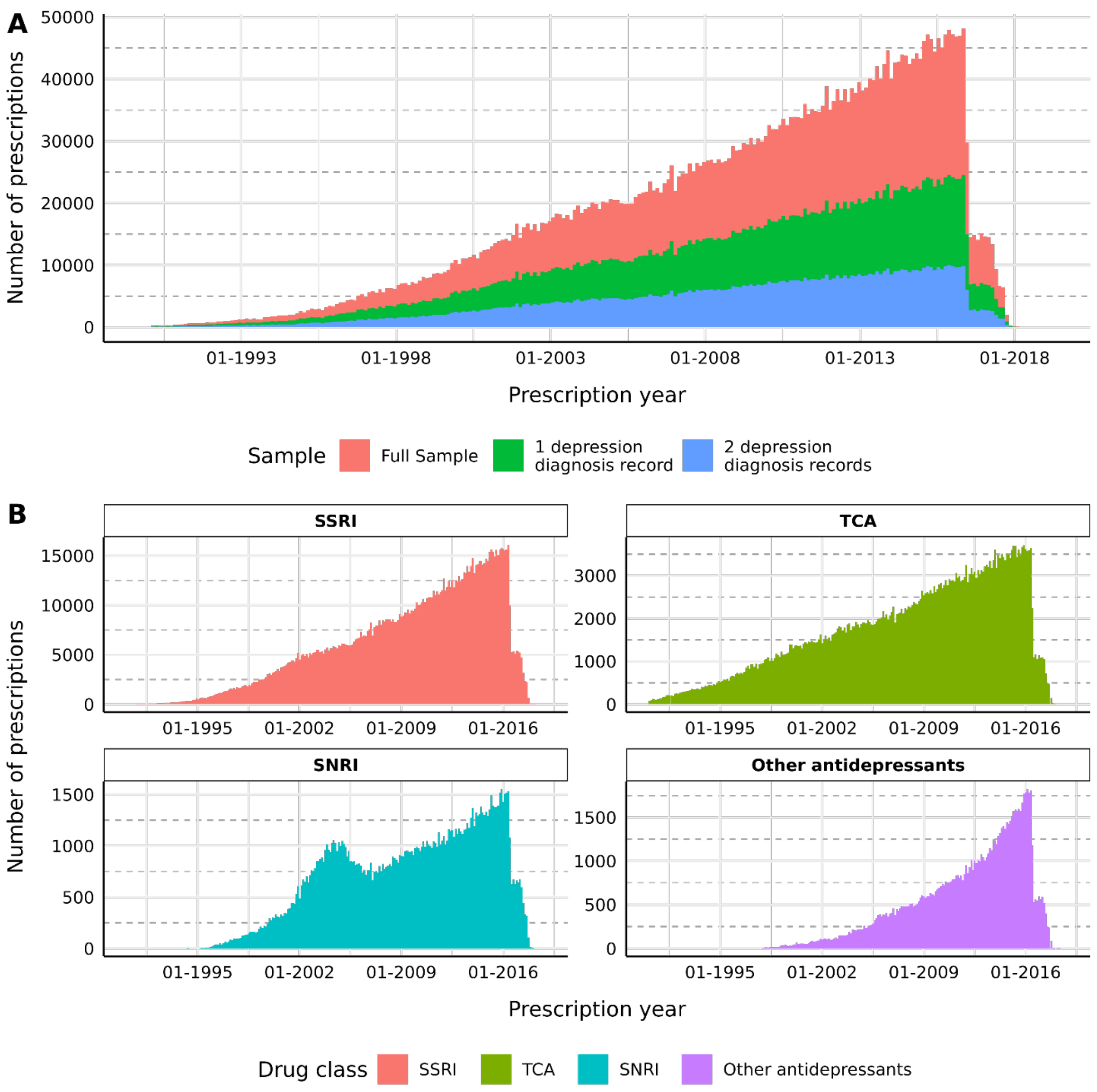

Caption

(A) Number of prescriptions across time stratified by sample; **(B)** Number of prescriptions in full primary sample stratified by 4 antidepressant classes with most prescriptions.

Histogram constructed by dividing prescriptions into 250 bins according to date of prescription.

The decrease in SSRI prescriptions in 2016 is due to different data extraction dates for different providers of EHR systems to UKB, ranging from August 2016 to July 2017.

Abbreviations

SNRI = serotonin–norepinephrine reuptake inhibitors; SSRI = selective serotonin reuptake inhibitors; TCA = tricyclic antidepressants.

Supplementary figure 4. Distribution plot for time to switch for SSRI switchers in primary sample, UK Biobank

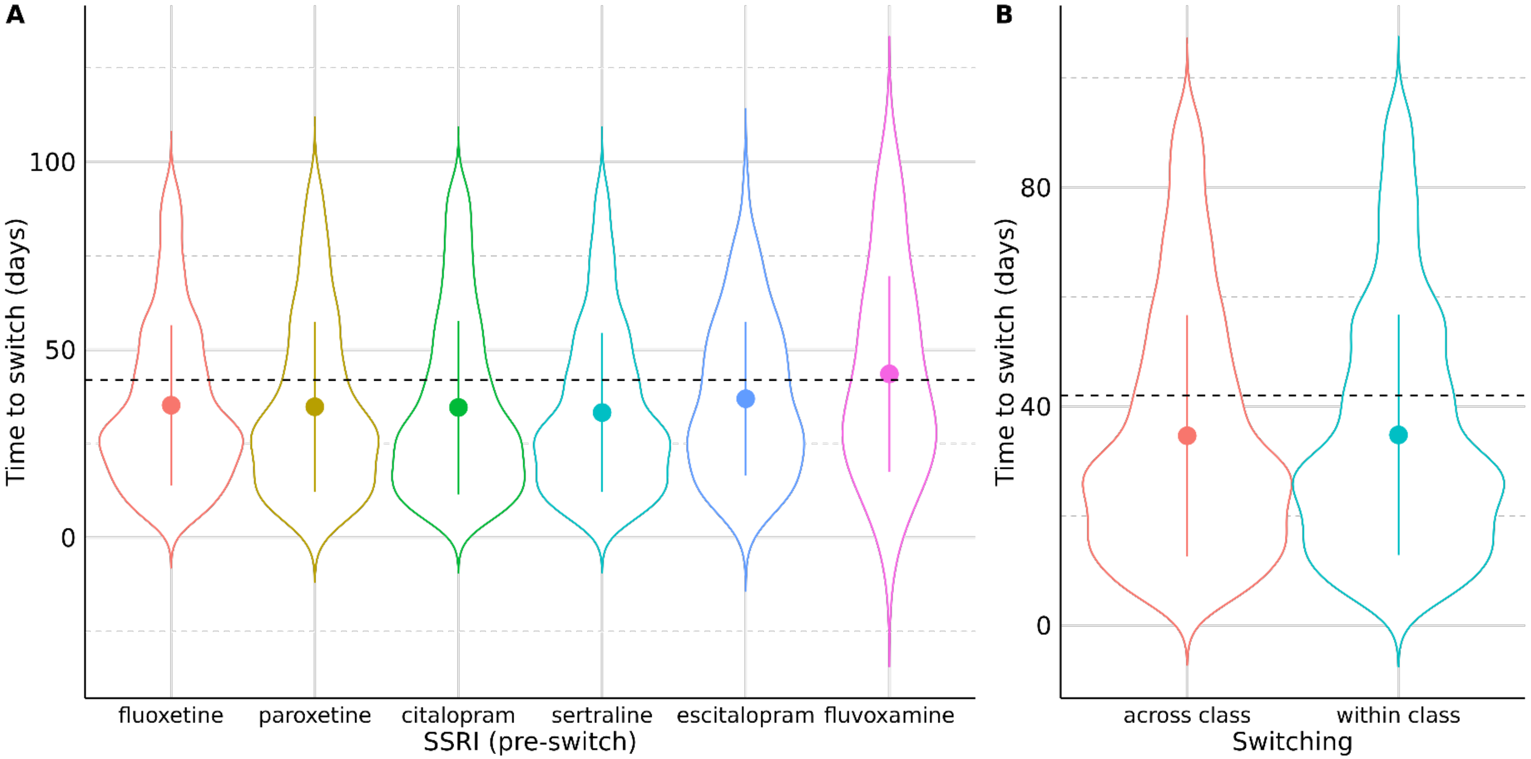

Caption

Distribution plots for **(A)** index SSRI before switching; **(B)** comparison between across-class (other drug classes) and within-class switches (to another SSRI). Dashed lines (black) represent 42 days (6 weeks) from index prescription of SSRI.

Abbreviations

SSRI = selective serotonin reuptake inhibitors.

Supplementary figure 5. Distribution of index SSRI used and age at index date for SSRI prescription of primary sample, UK Biobank

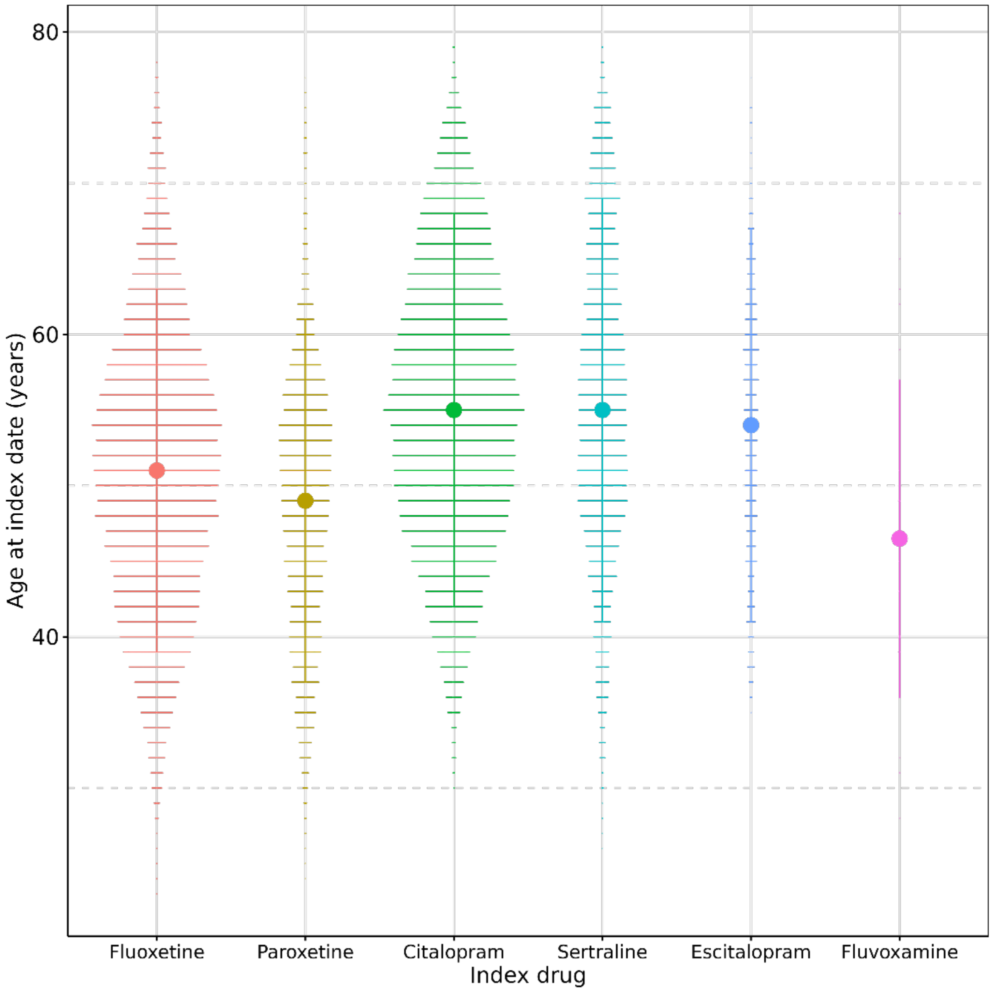

Caption

Index drug defined as: (1) switchers: SSRI that is switched subsequently; (2) non-switchers: SSRI with ≥ 3 consecutive prescriptions.

Abbreviations

SSRI = selective serotonin reuptake inhibitors.

Supplementary figure 6. Sex-specific descriptive analysis for primary sample, UK Biobank

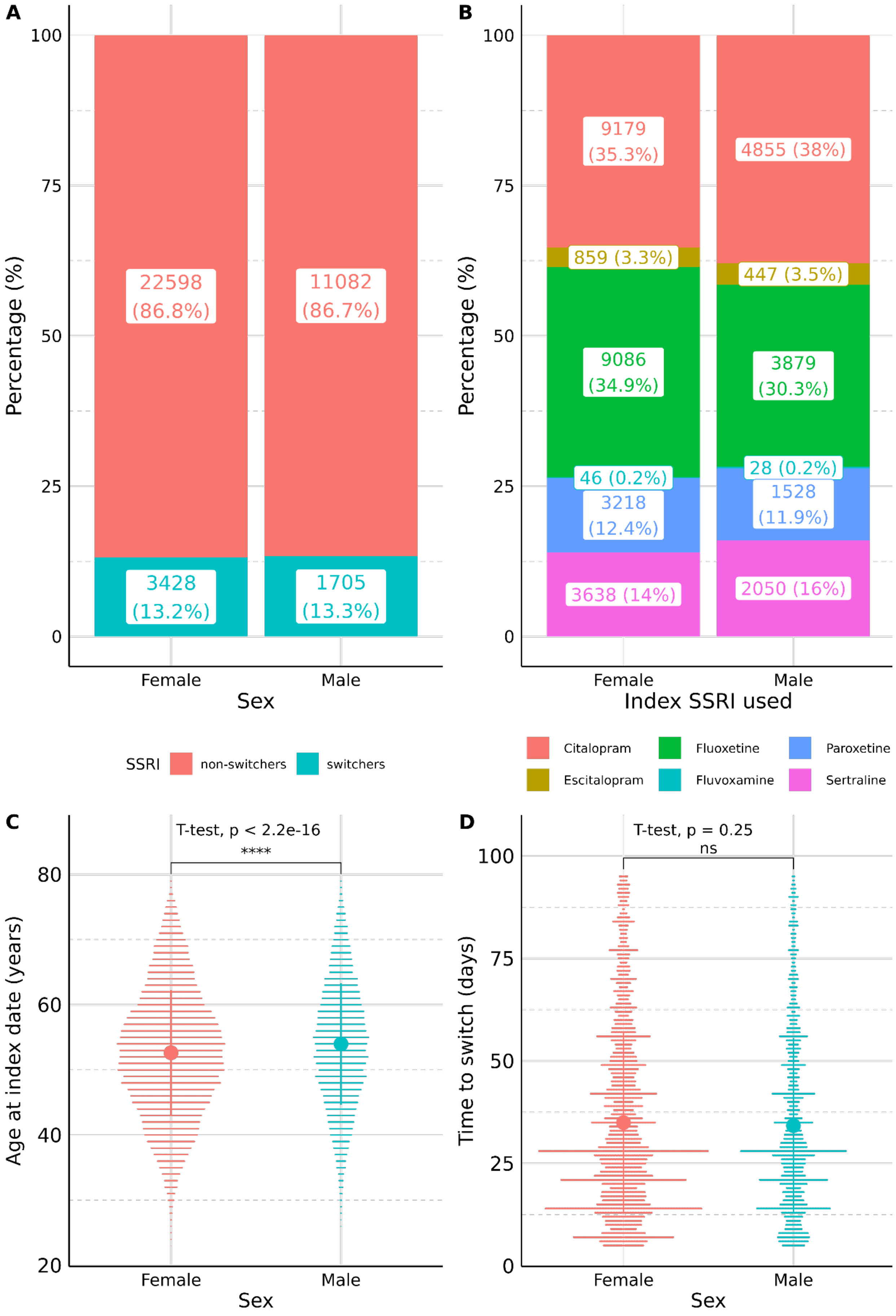

Caption

Sex-specific analysis for **(A)** proportion of switchers; **(B)** index SSRI used; **(C)** age at index date (years); **(D)** time to switch (days).

Proportion of switchers and index SSRI used expressed in count (percentage).

Statistical significance for gender-specific differences in mean age at index date and time to switch assessed by T-test (two-sided).

Abbreviations

ns = not-significant; SSRI = selective serotonin reuptake inhibitors.

Supplementary figure 7. Alluvial plot for switching patterns in Generation Scotland replication sample

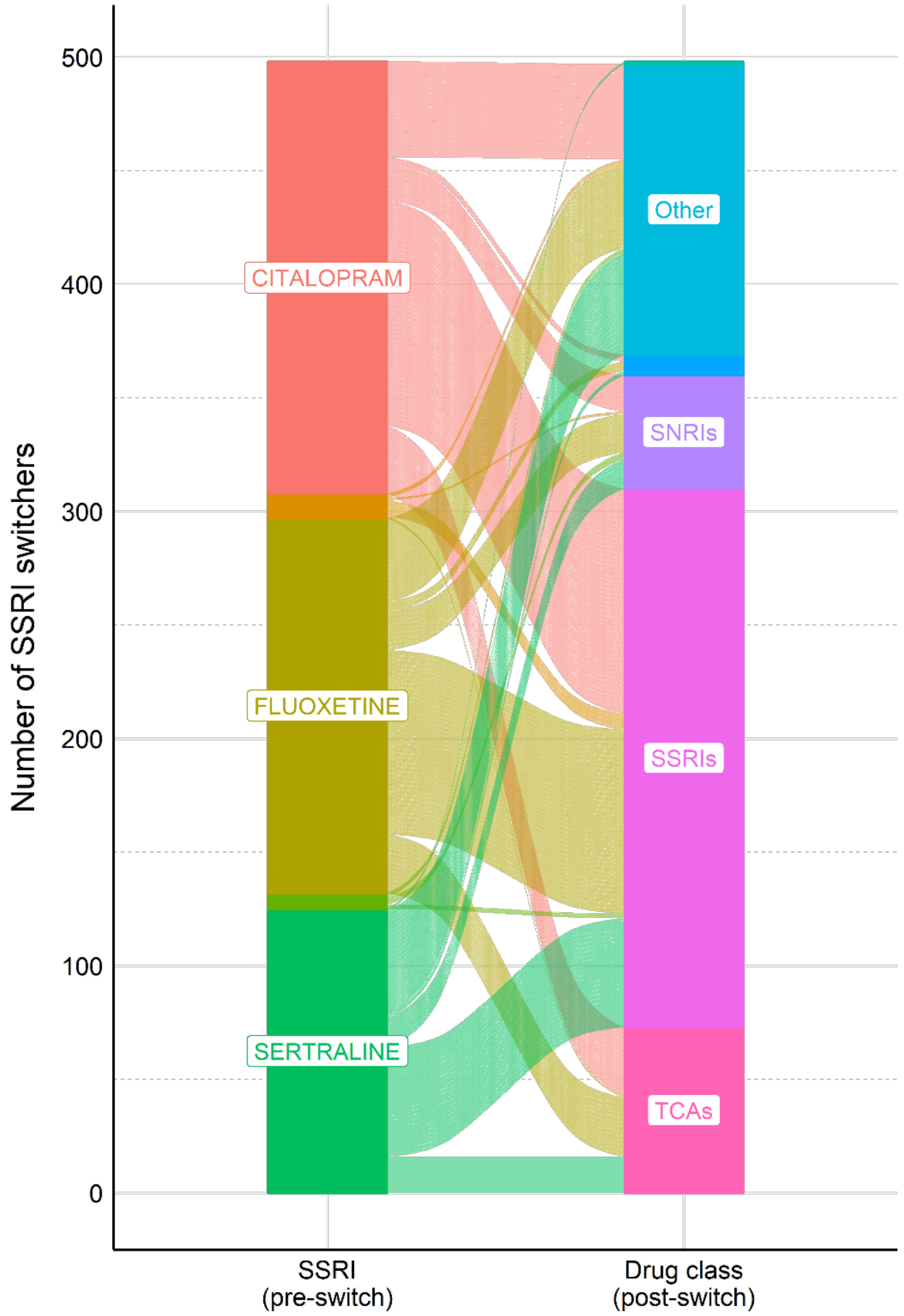

Abbreviations

SNRIs = serotonin–norepinephrine reuptake inhibitors; SSRIs = selective serotonin reuptake inhibitors; TCAs = tricyclic antidepressants.

Supplementary figure 8: Distribution of switch patterns across assessment centres

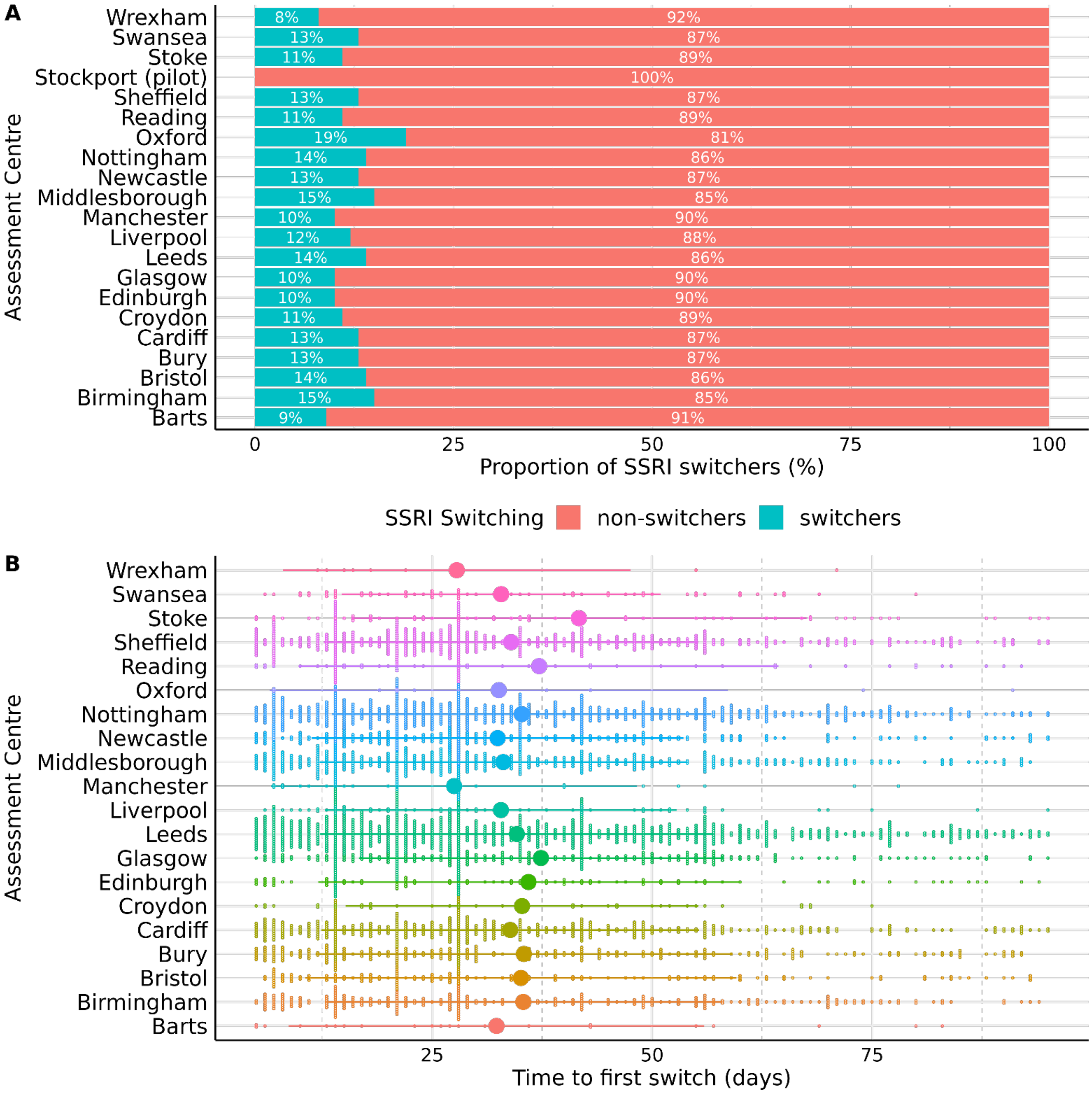

Abbreviations

SSRI = selective serotonin reuptake inhibitors.

Supplementary figure 9. Relationship between switching patterns and SSRI index date percentiles in UKB

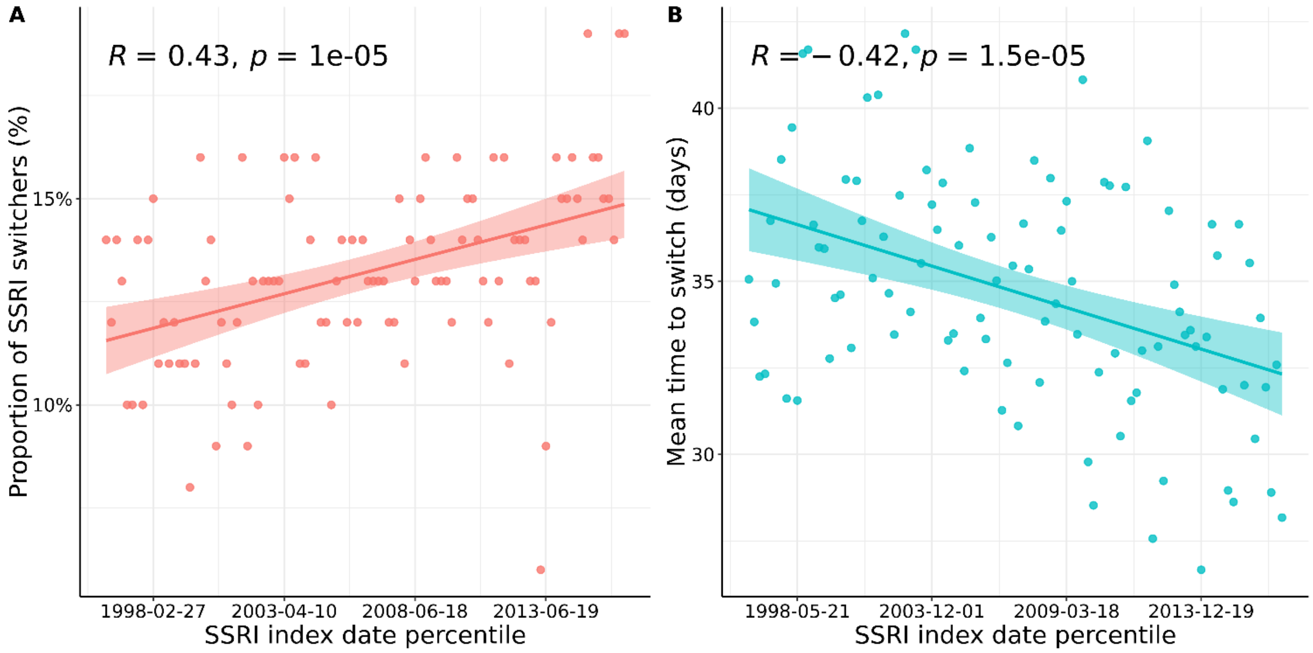

Caption

Correlation plots between **(A)** proportion of SSRI switchers within each percentile; **(B)** mean time to switch (days) within each percentile, and index date percentile of SSRI.

Abbreviations

SSRI = selective serotonin reuptake inhibitors.

Supplementary figure 10. BMI distribution at assessment for primary sample, UK Biobank

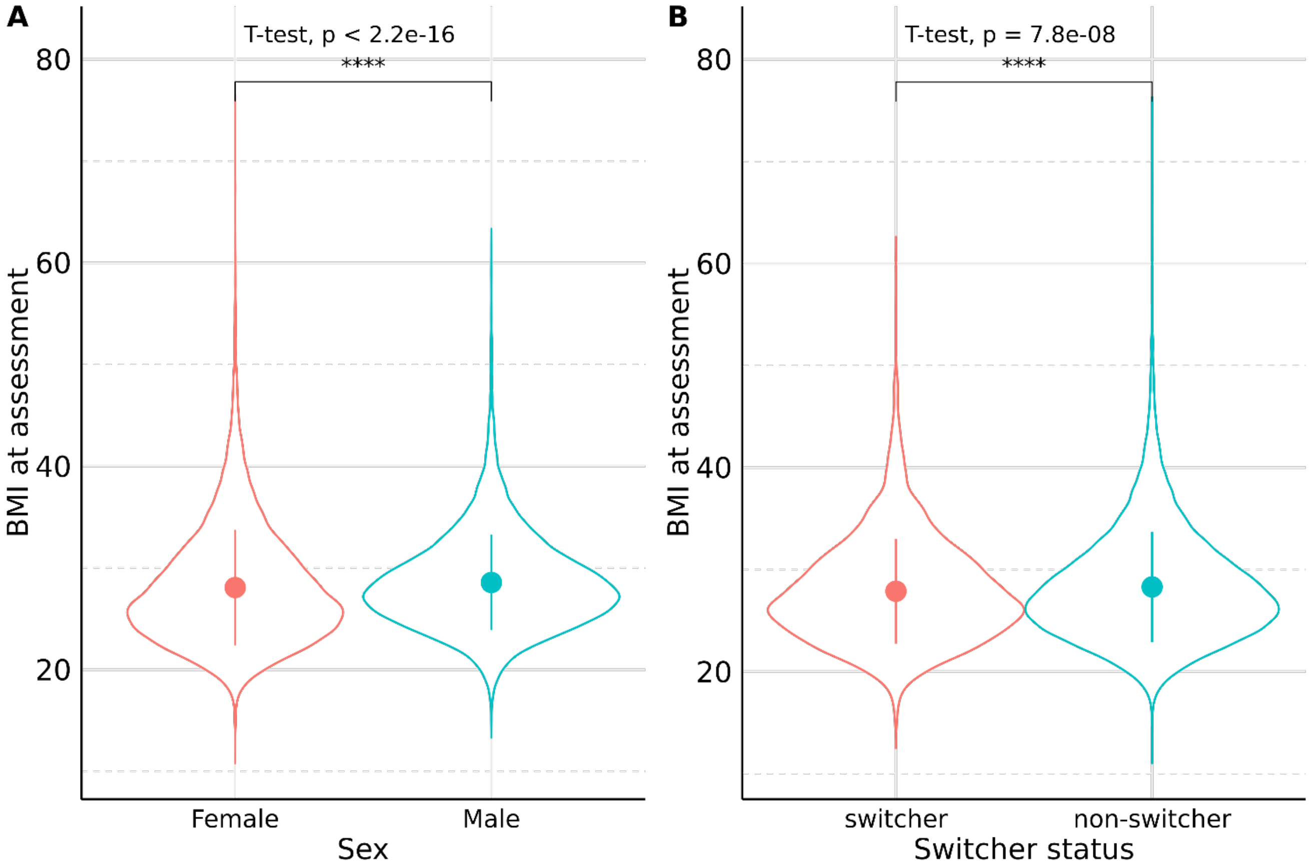

Caption

Statistical significance in mean between groups assessed by two-sided t-test.

Dots and error bars represent mean and standard deviations of distributions respectively.

Abbreviations

BMI = body mass index.

Supplementary figure 11: Distribution of neuroticism scores at assessment for primary sample

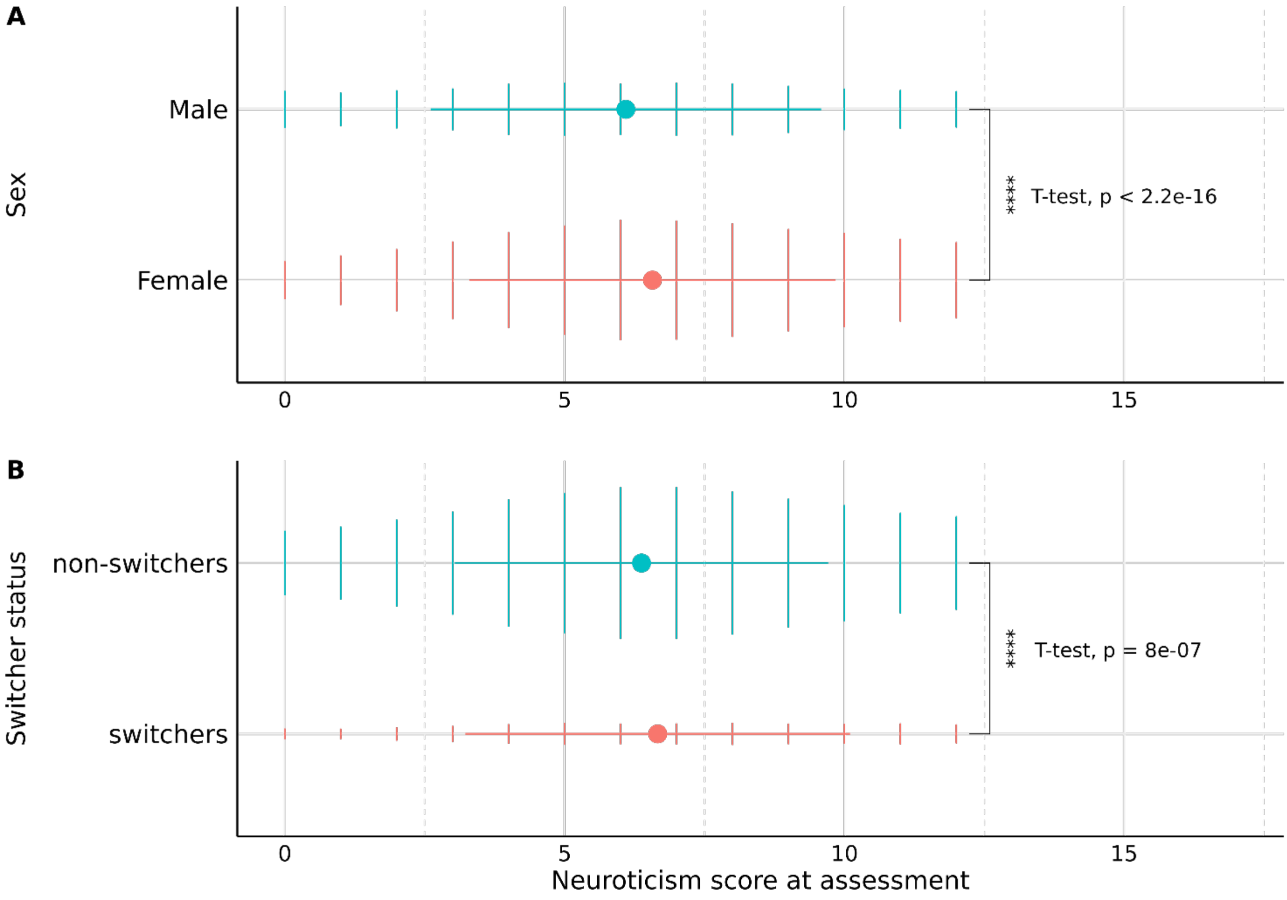

Caption

Statistical significance in mean between groups assessed by two-sided t-test.

Dots and error bars represent mean and standard deviations of distributions respectively.

Supplementary figure 12. Distribution of demographic variables across assessment centres

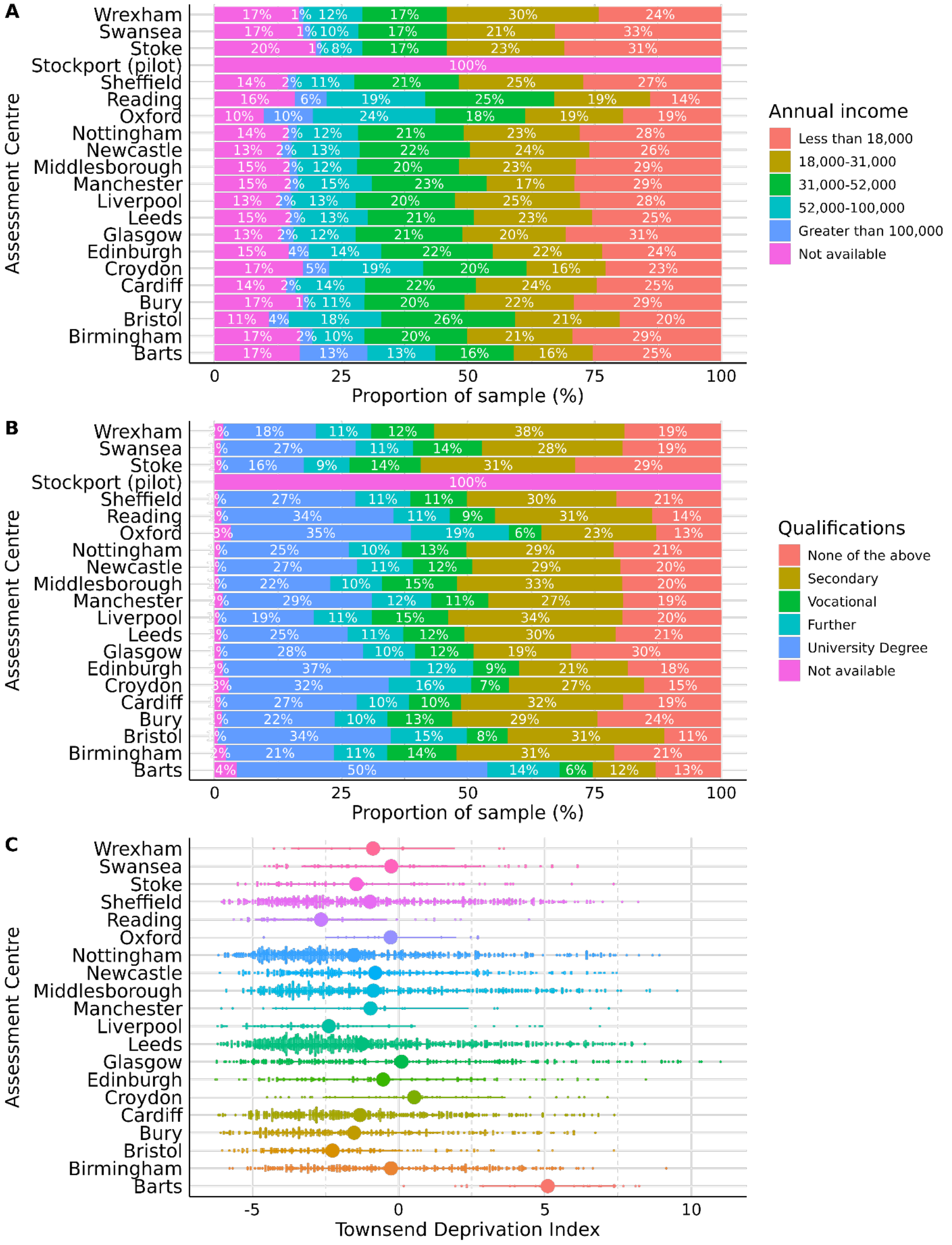

Supplementary figure 13: GCTB estimates on *Pi* (polygenicity) and *S* (negative selection)

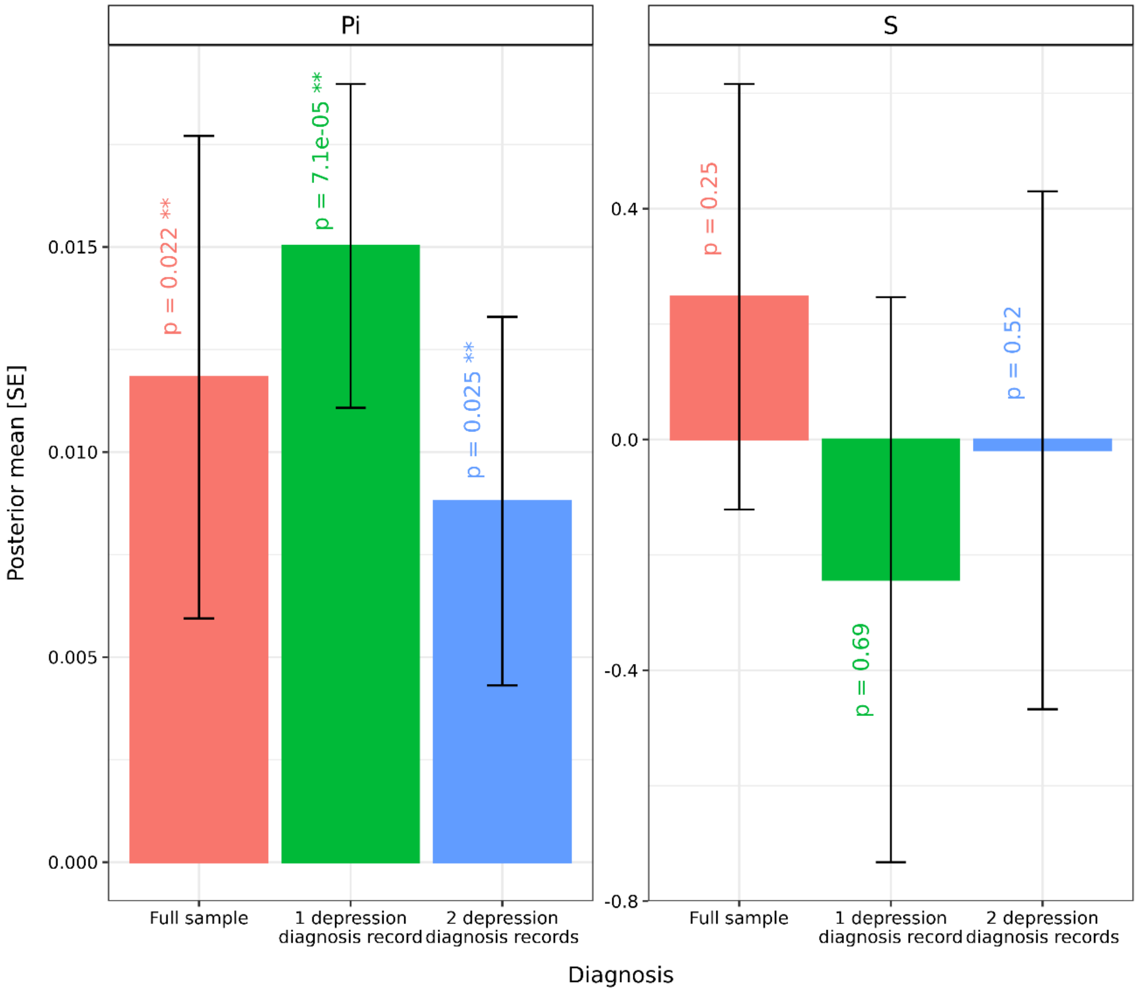

Caption

GCTB estimates on number of Pi (degree of polygenicity) and S (value for natural selection), expressed as posterior mean (standard error).

Abbreviations

GCTB = Genome-wide Complex Trait Bayesian; SE = standard error.

Supplementary figure 14. Heritability distribution for GCTB per iteration in MCMC sampler

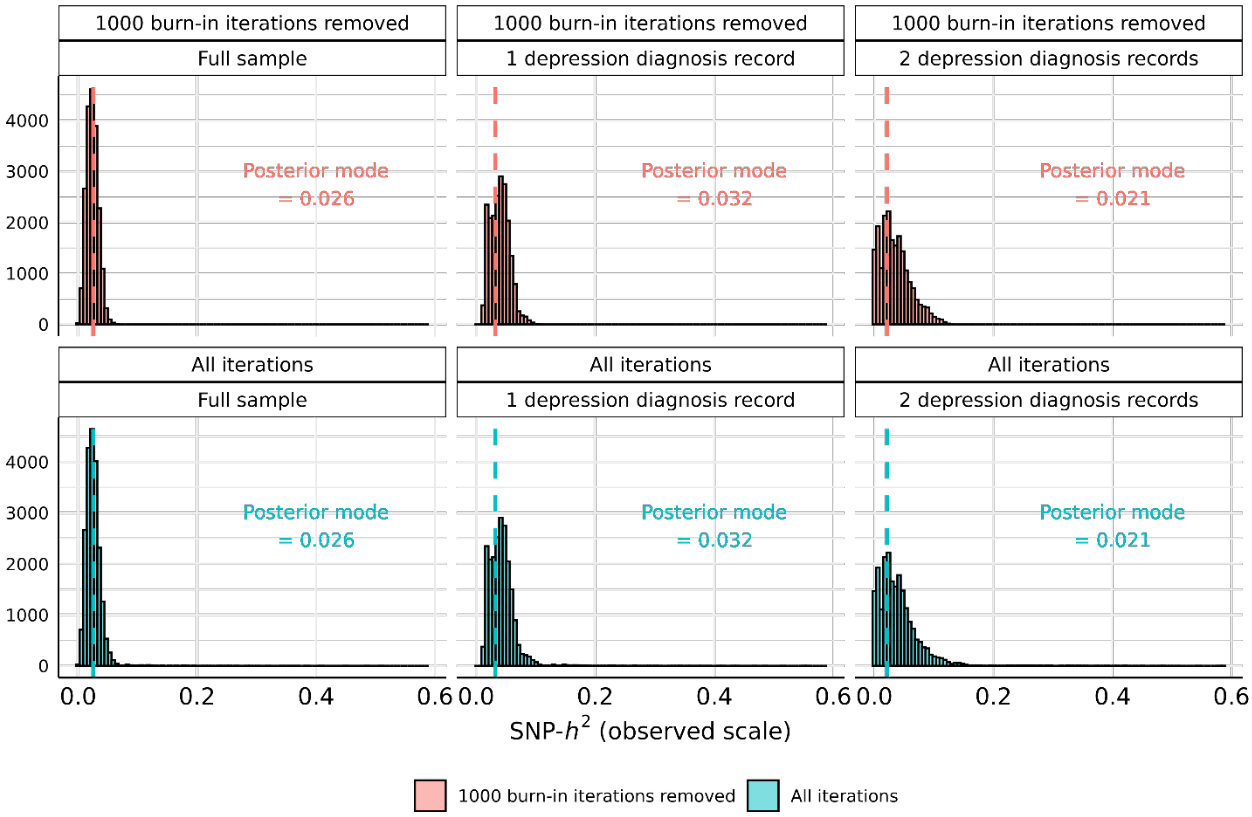

Caption

Heritability estimates divided into 100 bins for visualization. 21000 iterations (with 1000 burn-in iterations removed) were used as the options for MCMC sampling in GCTB estimation of heritability.

Abbreviations

GCTB = Genome-wide Complex Trait Bayesian; h^2 = heritability; MCMC = Markov chain Monte Carlo; SNP = single-nucleotide polymorphism.

Supplementary figure 15. Manhattan plot for SSRI switching (full sample)

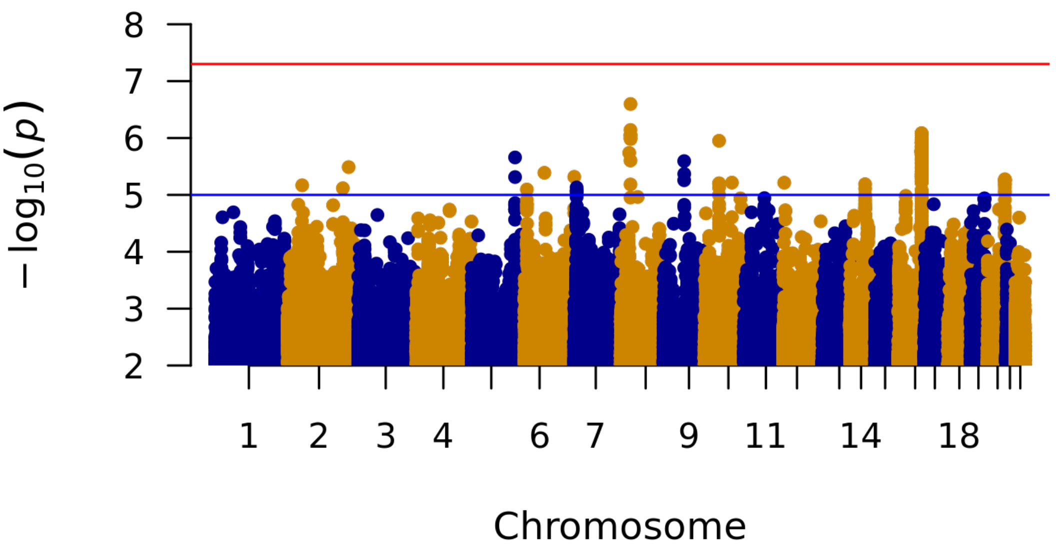

Caption

Red line represents genome-wide significance ($p < 5 \times 10^{-8}$). Blue line represents suggestive hits ($p < 1 \times 10^{-5}$).

Abbreviations

SSRI = selective serotonin reuptake inhibitors.

Supplementary figure 16: Manhattan plot for SSRI switching (≥ 1 depression diagnosis record)

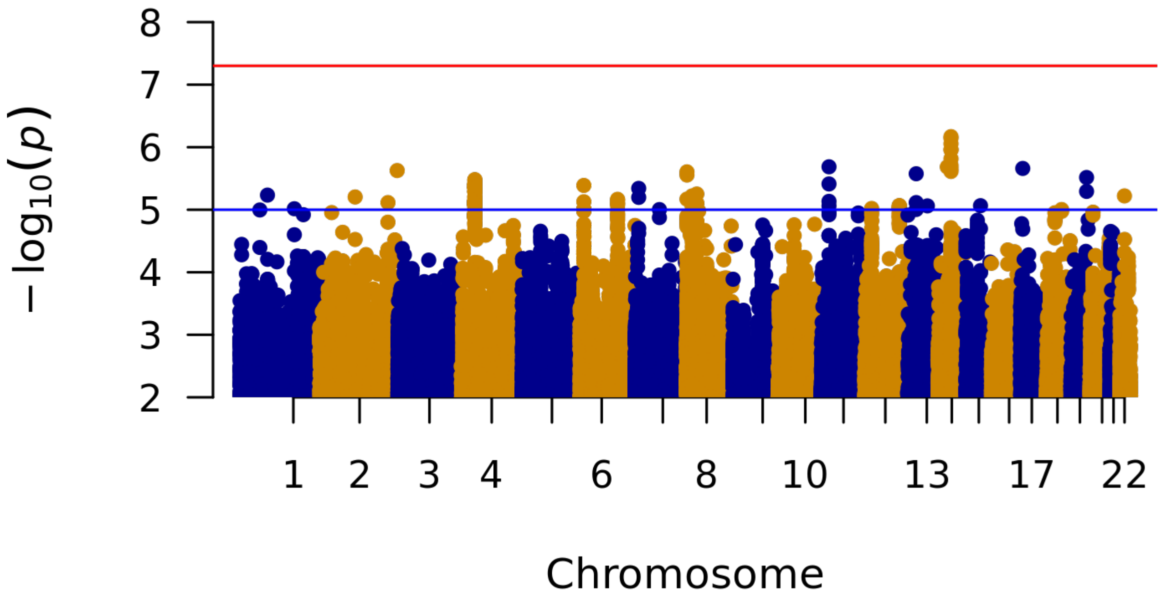

Caption

Red line represents genome-wide significance ($p < 5 \times 10^{-8}$). Blue line represents suggestive hits ($p < 1 \times 10^{-5}$).

Abbreviations

MDD = major depressive disorder; SSRI = selective serotonin reuptake inhibitors.

Supplementary figure 17: Manhattan plot for SSRI switching (≥ 2 depression diagnosis records)

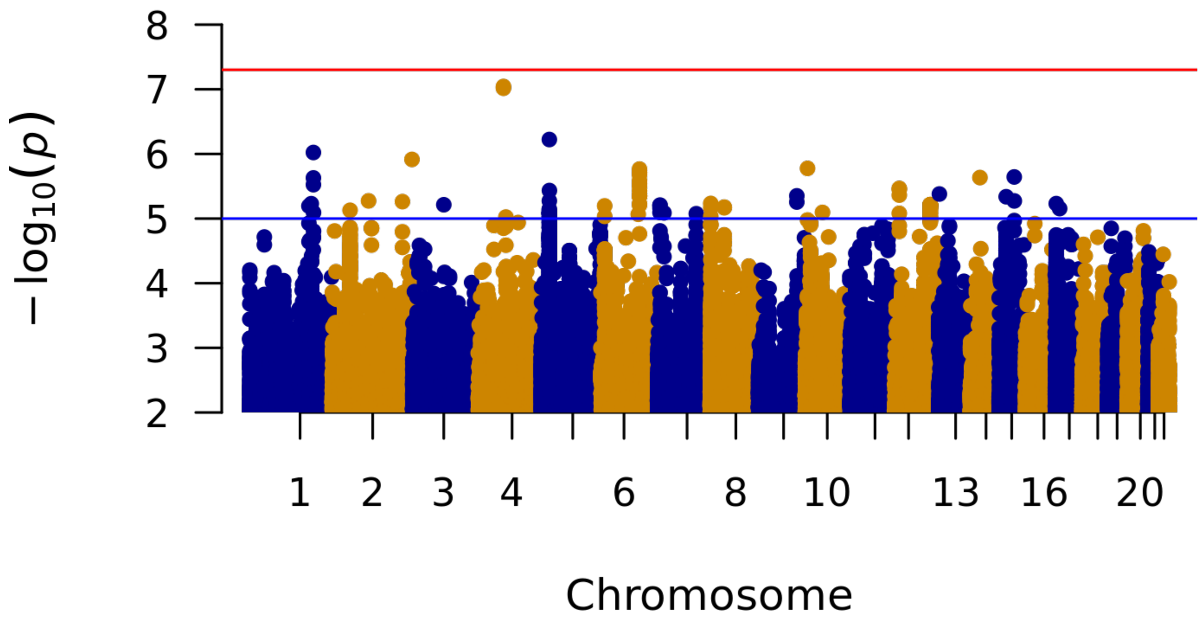

Caption

Red line represents genome-wide significance ($p < 5 \times 10^{-8}$). Blue line represents suggestive hits ($p < 1 \times 10^{-5}$).

Abbreviations

MDD = major depressive disorder; SSRI = selective serotonin reuptake inhibitors.

Supplementary figure 18. QQ plot for SSRI switching (full sample)

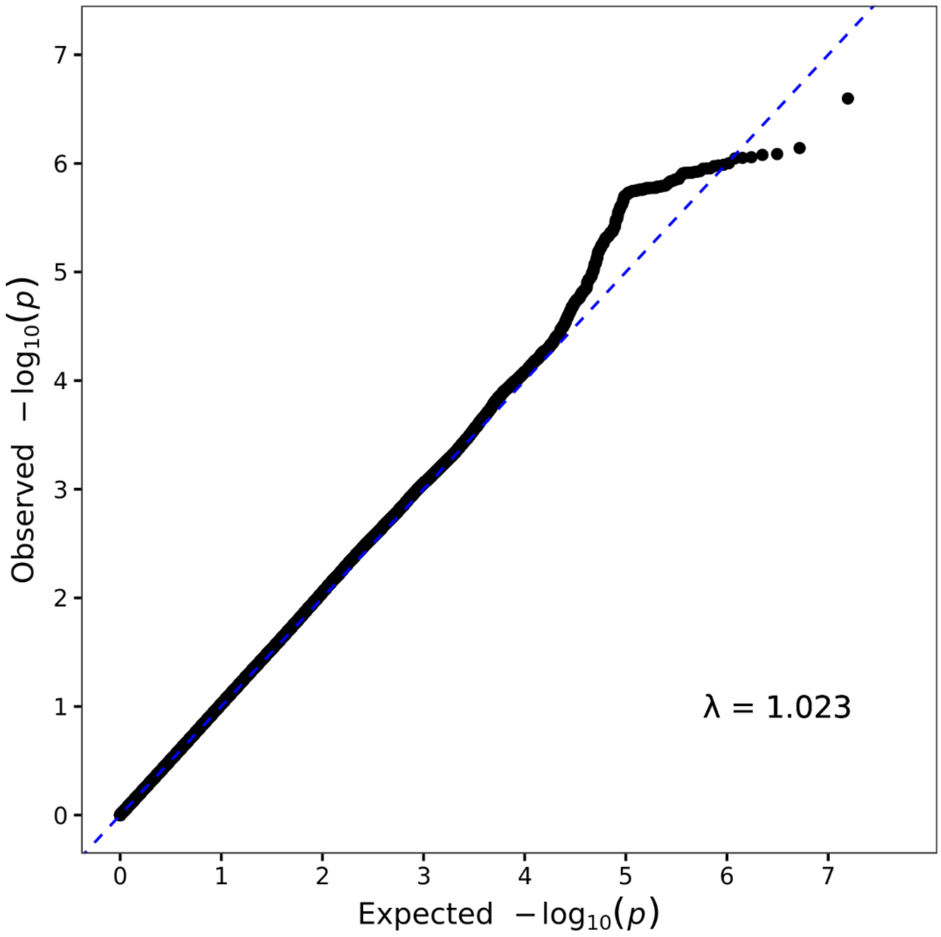

Abbreviations

λ = lambda for genomic control; MDD = major depressive disorder; SSRI = selective serotonin reuptake inhibitors.

Supplementary figure 19. QQ plot for SSRI switching (≥ 1 depression diagnosis record)

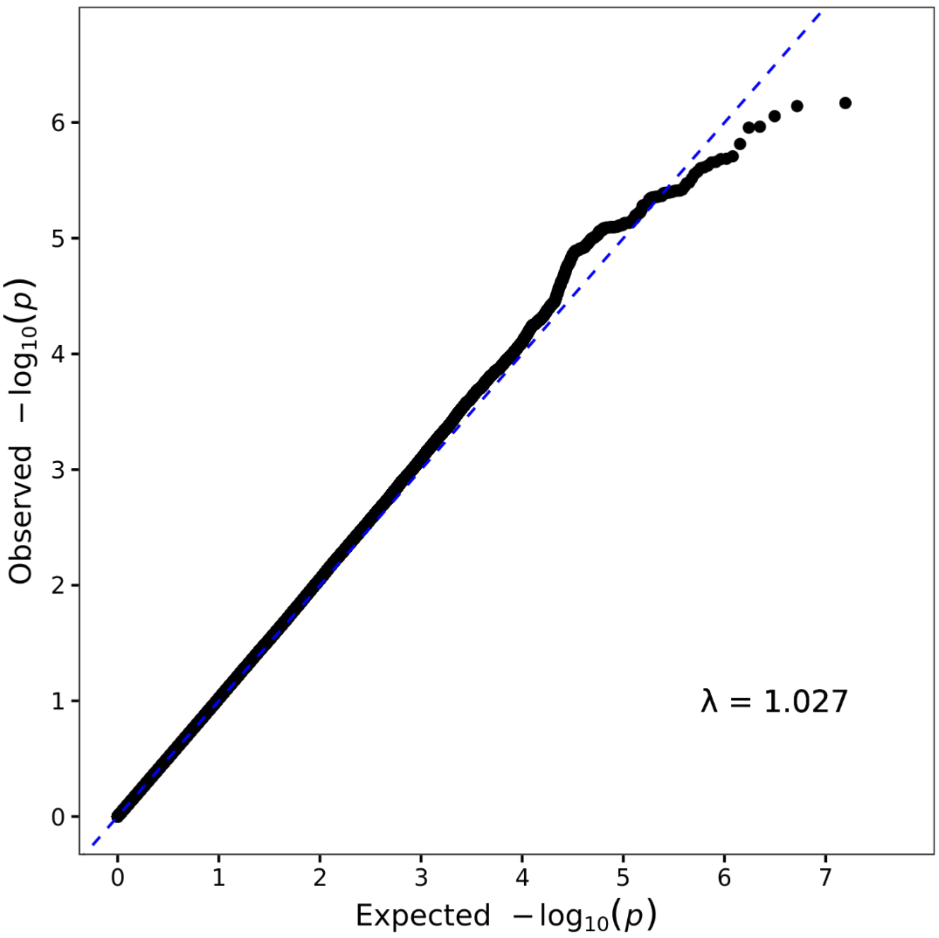

Abbreviations

λ = lambda for genomic control; MDD = major depressive disorder; SSRI = selective serotonin reuptake inhibitors.

Supplementary figure 20. QQ plot for SSRI switching (≥ 2 depression diagnosis records)

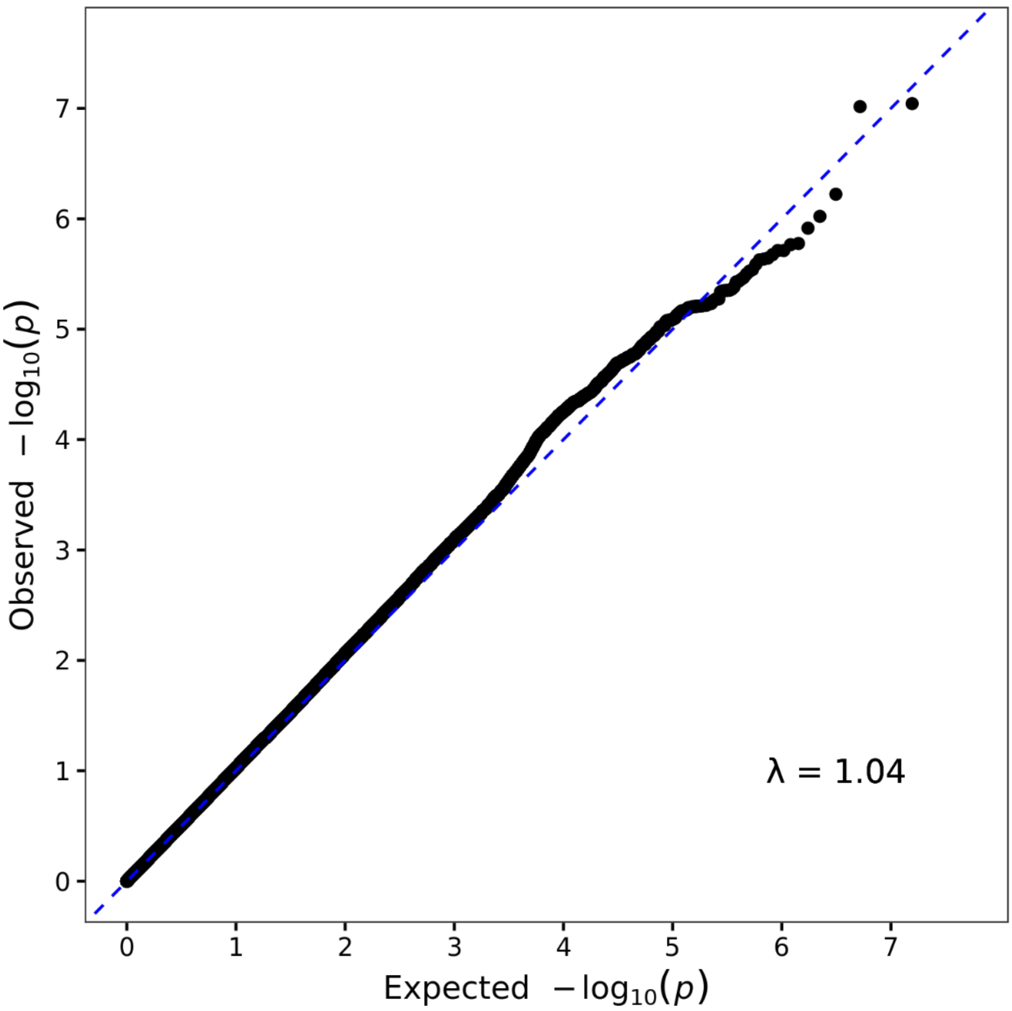

Abbreviations

λ = lambda for genomic control; MDD = major depressive disorder; SSRI = selective serotonin reuptake inhibitors.

References

1. Bycroft C, Freeman C, Petkova D, Band G, Elliott LT, Sharp K, *et al.* (2018): The UK Biobank resource with deep phenotyping and genomic data. *Nature* 562: 203–209.
2. Davis KAS, Coleman JRI, Adams M, Allen N, Breen G, Cullen B, *et al.* (2020): Mental health in UK Biobank - development, implementation and results from an online questionnaire completed by 157 366 participants: a reanalysis. *BJPsych Open* 6: e18.
3. McCarthy S, Das S, Kretzschmar W, Delaneau O, Wood AR, Teumer A, *et al.* (2016): A reference panel of 64,976 haplotypes for genotype imputation. *Nat Genet* 48: 1279–1283.
4. Huang J, Howie B, McCarthy S, Memari Y, Walter K, Min JL, *et al.* (2015): Improved imputation of low-frequency and rare variants using the UK10K haplotype reference panel. *Nat Commun* 6: 8111.
5. Mbatchou J, Barnard L, Backman J, Marcketta A, Kosmicki JA, Ziyatdinov A, *et al.* (2021): Computationally efficient whole-genome regression for quantitative and binary traits. *Nat Genet* 53: 1097–1103.
6. The UK Biobank (2022, September 26): Health-related outcomes data. Retrieved June 26, 2023, from https://www.ukbiobank.ac.uk/enable-your-research/about-our-data/health-related-outcomes-data
7. Fabbri C, Hagenaars SP, John C, Williams AT, Shrine N, Moles L, *et al.* (2021): Genetic and clinical characteristics of treatment-resistant depression using primary care records in two UK cohorts. *Mol Psychiatry* 26: 3363–3373.
8. Milbourn H, McCartney D, Richmond A, Campbell A, Flaig R, Robertson S, *et al.* (2024): Generation Scotland: an update on Scotland's longitudinal family health study. *BMJ Open* 14: e084719.

9. Habota T, Sandu A-L, Waiter GD, McNeil CJ, Steele JD, Macfarlane JA, *et al.* (2019): Cohort profile for the STratifying Resilience and Depression Longitudinally (STRADL) study: A depression-focused investigation of Generation Scotland, using detailed clinical, cognitive, and neuroimaging assessments. *Wellcome Open Res* 4: 185.

10. Ge T, Chen C-Y, Ni Y, Feng Y-CA, Smoller JW (2019): Polygenic prediction via Bayesian regression and continuous shrinkage priors. *Nat Commun* 10: 1776.

11. Pain O, Al-Chalabi A, Lewis CM (2024): The GenoPred Pipeline: A Comprehensive and Scalable Pipeline for Polygenic Scoring. *Bioinformatics* btae551.

12. Pain O, Hodgson K, Trubetskoy V, Ripke S, Marshe VS, Adams MJ, *et al.* (2022): Identifying the Common Genetic Basis of Antidepressant Response. *Biol Psychiatry Glob Open Sci* 2: 115–126.

13. Wray NR, Ripke S, Mattheisen M, Trzaskowski M, Byrne EM, Abdellaoui A, *et al.* (2018): Genome-wide association analyses identify 44 risk variants and refine the genetic architecture of major depression. *Nat Genet* 50: 668–681.

14. Trubetskoy V, Pardiñas AF, Qi T, Panagiotaropoulou G, Awasthi S, Bigdeli TB, *et al.* (2022): Mapping genomic loci implicates genes and synaptic biology in schizophrenia. *Nature* 604: 502–508.

15. Manichaikul A, Mychaleckyj JC, Rich SS, Daly K, Sale M, Chen W-M (2010): Robust relationship inference in genome-wide association studies. *Bioinformatics* 26: 2867–2873.

16. The International HapMap 3 Consortium (2010): Integrating common and rare genetic variation in diverse human populations. *Nature* 467: 52–58.

17. 1000 Genomes Project Consortium, Auton A, Brooks LD, Durbin RM, Garrison EP, Kang HM, *et al.* (2015): A global reference for human genetic variation. *Nature* 526: 68–74.

18. Yang J, Lee SH, Goddard ME, Visscher PM (2011): GCTA: a tool for genome-wide complex trait analysis. *Am J Hum Genet* 88: 76–82.

19. Zeng J, Xue A, Jiang L, Lloyd-Jones LR, Wu Y, Wang H, *et al.* (2021): Widespread signatures of natural selection across human complex traits and functional genomic categories. *Nat Commun* 12: 1164.

20. Zeng J, de Vlaming R, Wu Y, Robinson MR, Lloyd-Jones LR, Yengo L, *et al.* (2018): Signatures of negative selection in the genetic architecture of human complex traits. *Nat Genet* 50: 746–753.

21. Meredith M, Kruschke J (2022): HDInterval: Highest (Posterior) Density Intervals, version 0.2.4. Retrieved from https://CRAN.R-project.org/package=HDInterval
